# Atomistic fingerprint of hyaluronan–CD44 binding

**DOI:** 10.1371/journal.pcbi.1005663

**Published:** 2017-07-17

**Authors:** Joni Vuorio, Ilpo Vattulainen, Hector Martinez-Seara

**Affiliations:** 1 Department of Physics, Tampere University of Technology, Tampere, Finland; 2 Department of Physics, University of Helsinki, Helsinki, Finland; 3 MEMPHYS - Centre for Biomembrane Physics, University of Southern Denmark, Odense, Denmark; 4 Institute of Organic Chemistry and Biochemistry, The Czech Academy of Sciences, Prague, Czech Republic; Baltimore, UNITED STATES

## Abstract

Hyaluronan is a polyanionic, megadalton-scale polysaccharide, which initiates cell signaling by interacting with several receptor proteins including CD44 involved in cell-cell interactions and cell adhesion. Previous studies of the CD44 hyaluronan binding domain have identified multiple widespread residues to be responsible for its recognition capacity. In contrast, the X-ray structural characterization of CD44 has revealed a single binding mode associated with interactions that involve just a fraction of these residues. In this study, we show through atomistic molecular dynamics simulations that hyaluronan can bind CD44 with three topographically different binding modes that in unison define an interaction fingerprint, thus providing a plausible explanation for the disagreement between the earlier studies. Our results confirm that the known crystallographic mode is the strongest of the three binding modes. The other two modes represent metastable configurations that are readily available in the initial stages of the binding, and they are also the most frequently observed modes in our unbiased simulations. We further discuss how CD44, fostered by the weaker binding modes, diffuses along HA when attached. This 1D diffusion combined with the constrained relative orientation of the diffusing proteins is likely to influence the aggregation kinetics of CD44. Importantly, CD44 aggregation has been suggested to be a possible mechanism in CD44-mediated signaling.

## Author summary

Hyaluronan is a natural sugar polymer in our bodies. Besides acting as a space-filling agent for example in multiple connective tissues, it also functions as a cellular cue in cancer and inflammation. Our tissues sense hyaluronan through receptors—proteins that sit at the surface of cells and grab the molecules they are expected to recognize. Although the knowledge associated with hyaluronan and its receptors is constantly accumulating, the molecular-level insight is largely missing or incomplete due to the lack of techniques able to probe the dynamics of protein–carbohydrate interactions with sufficiently high resolution. In this work, we characterize the binding of hyaluronan to its receptor CD44 with atomistic precision. We achieve this level of precision by employing atomistic molecular dynamics simulations. This computational technique allows one to follow the movement of atoms of a virtual system at scales beyond the resolution of any experimental technique. Our work specifically focuses on the different stages of hyaluronan–CD44 binding, and we observe the process to involve three different binding modes, making it more versatile than previously thought. Our insights, therefore, promote the understanding of the interplay between hyaluronan and HA, thereby fostering development of new drugs or inhibitors to malignancies, such as cancer metastasis.

## Introduction

Hyaluronic acid (HA) also known as hyaluronan is a natural carbohydrate polymer constituted by a repeating disaccharide of glucuronic acid (GlcUA) and *N*-acetylglucosamine (GlcNAc) ([-β(1,4)-GlcUA-β(1,3)-GlcNAc-]_n_) [1]. Reaching molecular weights of up to 10^6^ Da (i.e., several thousand disaccharides), HA acts as a space-filling agent, molecular lubricant, and cell migration promoter in processes such as leukocyte trafficking, modulating embryonic morphogenesis and tumor metastasis. It is also an integral component of both the extracellular and pericellular matrices, where it interacts with cells through HA binding proteins [2], particularly with CD44 [3].

CD44 is a type I transmembrane receptor protein, with HA as its main ligand. It is expressed in a wide variety of human cell types, including leukocytes, endothelial cells, and fibroblasts [3]. Structurally, the canonical form of human CD44 consists of 723 residues, divided into four distinct domains: the extracellular HA binding and stalk domains, the transmembrane domain, and the cytosolic region. From these, only the 158-residue HA binding domain (HABD) has been structurally characterized [4]. As its name implies, the majority of the HA-binding capacity of CD44 stems from the globular HABD which can even be expressed as an individual soluble protein that retains its ability to bind HA [5]. It is composed of a link module, which is extended by additional N-terminal and C-terminal flanking regions, that together form a globular HA-binding unit stabilized by three disulfide bridges [4]. The link module itself is a conserved α/β-fold shared by other similar HA binding proteins, such as TSG-6 [6] and LYVE-1 [7].

The CD44-HABD has been studied extensively for two decades [8, 9]. In addition to a high number of experiments concentrating on its pathophysiology, numerous studies have also focused on the molecular level details of its structure and function [4, 10–14]. The goal has been to fully understand the factors affecting the CD44-HABD–HA interplay. On the one hand, HABD can recognize HA from other carbohydrates, rendering their interaction highly specific [10]. On the other hand, HABD seems to possess the ability to regulate its affinity to HA, displaying clear differences in affinity between different cell types [15]. These changes in affinity have largely been attributed to N-glycosylations [16, 17] on the HABD surface, yet additional affinity-modifying mechanisms, such as conformational changes, do also exist [11, 12]. The binding ability may also be regulated in part by avidity-modifying mechanisms, such as the aggregation of the CD44 receptors [18, 19]. Similar regulation has been observed with LYVE-1 [20], an HA receptor homologous to CD44. Furthermore, the high molecular weight HA has been shown to induce the aggregation of CD44 receptors, while low molecular weight HA seems to lack this ability [21, 22].

Currently, there is one crystal structure of CD44-HABD in complex with HA [10]. It shows how the carbohydrate ligand binds to a shallow binding groove on the surface of the link module of HABD. This crystallographic binding is characterized by multiple specific hydrogen bonds along with a complementary surface topology of the protein, such as a small hydrophobic pocket able to accommodate the methyl group of a bound GlcNAc residue. The crystallographic study by Banerji et al. also shows two distinct conformations related to the nearby R41 side-chain. The ligand binds HABD with the same crystallographic binding mode in both conformations. In the so-called A-form, the R41 side-chain points outwards from the protein center, while in the B-form it is flipped towards the bound ligand, rendering direct hydrogen bonds possible with it. For this reason, the B-form has been suggested to represent the high-affinity conformation for HA binding [14], although the exact sequence of events related to the binding process remains unclear. The fact that the crystal structure of HABD without HA displayed only the A-form suggests that the B-form is stabilized by HA binding [4]. In any case, it is widely accepted that R41 is a particularly important residue in the recognition of HA, as mutating it to alanine completely abolishes HA binding [4, 8, 10, 23]. Its crucial role in the recognition has also been confirmed in multiple *in silico* assays [13, 14, 24].

While the study by Banerji et al. shows HA to bind exclusively to the binding groove on the link module [10], other studies, using both truncation and site-directed mutations, have identified binding residues outside the binding groove to be important for HA binding, too [8, 23]. For instance, the earliest attempt by Peach et al. [8] to map the HA binding surface of CD44 found multiple arginine and lysine residues located at two clusters—one in the link module (R29, K38, R41) and another in the C-terminal extension (R150, R154, K158, R162)—to be crucial for the binding. Especially the residues at the C-terminal extension pose an apparent conflict with the findings of Banerji et al., as they are structurally distant from the binding groove occupied by HA in the crystal structure. Some of the binding residues mapped to the link module, such as K38, are also located outside this binding groove, and therefore in conflict with the view proposed by the crystallographic study.

In another mutation assay, Bajorath et al. [23] found nine HABD residues to be important for HA binding. First, residues R41, Y42, R78, and Y79 located in the binding groove were found to be vital for HA binding, which agrees well with the crystallographic view. Second, additional residues outside the binding groove (K38, K68, N100, N101, and Y105) were identified as important for HA binding. Providing further support for these observations, two NMR assays recorded high chemical shift changes upon ligand binding in regions close to these residues [4, 25]. Overall, mapping all the identified binding residues onto the surface of HABD reveals a widespread interaction surface that cannot be covered by a single rod-like HA polymer.

Providing a partial explanation for the above dilemma, previous NMR experiments found a conformation shift in the C-terminal extension of HABD [4, 11, 12, 26]. This shift involves partial unfolding of the C-terminal flanking regions of HABD, thereby excluding the stable link module. In the ordered (O) conformation, the C-terminal β9 strand runs anti-parallel to β8, so that residues after β9 (158–169) go under the β7–β8 loop [11]. In the partially disordered (PD) conformation, the β9 strand unfolds and the orientation of the β8 strand changes with respect to the β0 strand. The existing crystal structures, such as the one resolved by Banerji et al., assume the O conformation [10], while several available NMR structures are seen in the PD form [4, 11].

Ogino et al. demonstrated that the C-terminus interconverts between these two conformations with an exchange rate of hundreds of milliseconds [12]. Furthermore, they also showed that this spontaneous conversion is not dependent on HA binding, although the PD conformation becomes more favorable in the ligand-bound form of the protein. This suggests that while crystallographic structures favor the ordered conformations independently of the presence of HA, NMR measurements in solution capture the PD conformation primarily when HA is present [12]. Recently, Favreau et al. employed the existing 3D structures and computer simulations to provide a structural reasoning for the preference of the PD conformation over the O conformation in the HA-bound case [14]. The conformational freedom gained by the C-terminal residues (R150, R154, K158, and R162) in the PD conformation allows them to attach to the bound ligand that would otherwise lie distant from these residues. This provides additional stabilization of the complex.

The O-to-PD transition gives an explanation as to why the C-terminal residues were seen important for HA binding in the earlier mutation assays. However, it does not explain why some residues outside the binding groove and also C-terminal extension have been identified to bind the ligand. The most distinct example of these residues is K38, identified as important for HA binding in multiple studies based both on alanine-scanning mutations and NMR chemical shifts [4, 8, 23]. Providing a complementary explanation to the wide-spread nature of the binding residues able to account for residues such as K38, Teriete et al. proposed the possibility of multiple different binding modes (i.e., CD44-HABD–HA interaction conformations) covering different regions of the HABD surface [4]. They speculated that in one mode HA could lie in the binding groove, while a second, upright mode could occupy a region perpendicular to the binding groove. It would extend from the C-terminus towards the β4–β5 loop, while passing through the region formed by R41, Y42, R78, and Y79.

Several structures of CD44 have been deposited to Protein Data Bank (PDB) [4, 10, 11, 27], triggering multiple computational simulation studies. These studies have shed light into the molecular level details of CD44 and its interaction with HA. For instance, one study reported a significant immobilization of the monosaccharide units of HA in the binding groove when bound in the crystallographic manner [28]. Water was also found to be severely restricted around the binding residues of HABD, in particular upon complexation with the ligand [29].

Three computational studies also focused on characterizing the A-to-B conformational transition observed in the crystal structures of HA-bound HABD. First, Jamison et al. conducted a comprehensive study elucidating the R41 side-chain dynamics and further characterizing the A-to-B conformational transition initially observed by Banerji et al. [13]. They also discovered that the A-to-B switch in the side-chain of R41 originates from a change in the ϕ backbone dihedral of the adjacent Y42. Meanwhile, Plazinski et al. elucidated the nature of the interactions in the crystal structure of HA_8_–CD44 complex. They came to the same conclusion that the A-to-B conformational transition stems from the ϕ backbone dihedral of Y42 [24]. Later, adaptive biasing force sampling was used by Favreau et al. to show that the B-form is energetically more favorable in the ligand-bound receptor compared to the A-form [14].

Two computational surveys also focused closely on the O-to-PD transition [14, 30]. First, Favreau et al. found the A-to-B and O-to-PD transitions to lack any allostery, and showed how the C-terminal extension gains freedom to bind HA in the PD conformation [14]. Second, Plazinski et al. probed the initial stages of the O-to-PD transition, using the umbrella sampling technique to measure the free energies of the uncoiling of the C-terminal end in wild-type CD44 and Y161A CD44 mutant [30]. This mutant has been observed to adapt exclusively to the PD conformation [12]. Yet, the results of Plazinski et al. showed only minimal differences between the wild-type and the mutant [30].

Finally, Faller et al. glycosylated HABD *in silico* at two N-glycosylation sites (N25 and N120) to probe the effect of N-glycans on the function of HABD [31]. They concluded that the negatively-charged sialic acids on the termini of the N-glycans charge paired with basic residues important for HA binding, such as R41 or R154. These sialic acids could thereby impede the binding of HA by competing for the same binding sites [31].

Despite providing valuable insight into the structure and dynamics of CD44-HABD, most of the aforementioned simulations lack the HA ligand entirely or sample only the crystallographic binding mode. Therefore, while providing detailed views of the studied processes, they have not sampled the complete binding process between CD44-HABD and HA.

**Fig 1:**
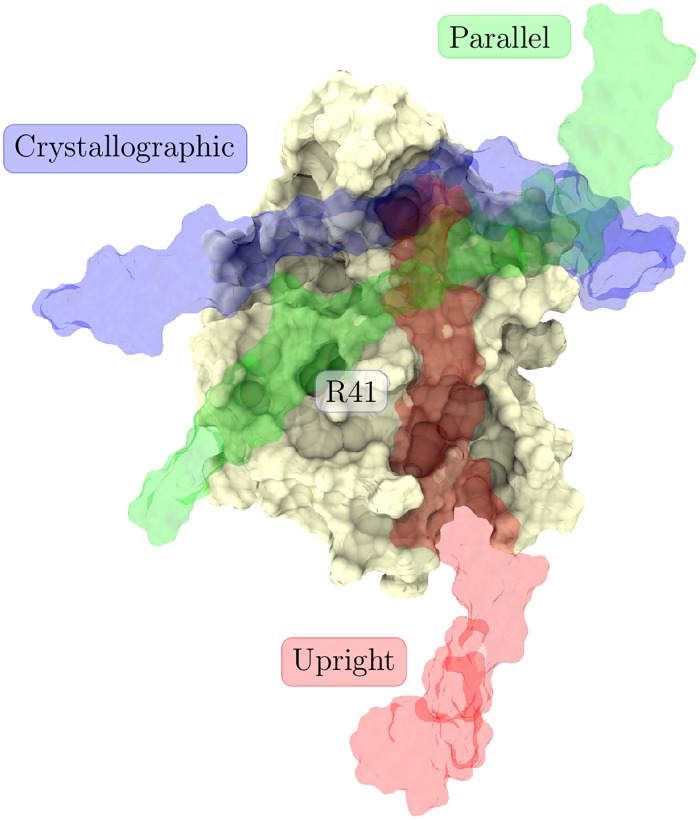
Three mutually exclusive binding modes. The three binding modes aligned together to the same CD44 template. Blue surface represents HA in the crystallographic mode (B-form, as in Ref. [10]), green represents the parallel mode, and red describes the upright binding mode (as in Ref. [4]). These orientations represent the situation in the beginning of the gathering simulations (Table A in S1 File).

In this work, we characterize, for the first time, all stages of the CD44–HA binding process. To this end, we performed different sets of atomistic molecular dynamics (MD) simulations that were designed to probe all the different stages on equal footing. To our surprise, the data revealed the existence of three well-defined binding modes (Fig 1):

1. “crystallographic” binding mode as described in Banerji et al. [10]
2. “parallel” binding mode, where the sugar rings of HA lie on the “lower” part of the binding groove (protein viewed as in Fig 1).
3. “upright” binding mode, where the HA binds the protein in an upright orientation, in a manner speculated previously by Teriete et al. [4] (protein viewed as in Fig 1).

Subsequent analysis of the simulation data shows that the crystallographic mode is the strongest of these three modes. However, it is not the most easily accessible and therefore not the most frequently binding mode at the initial stages of binding. The weaker modes foster the aggregation (in terms of kinetics) by fostering CD44 diffusion along HA. In this context, our findings provide a solid base to understand the molecular details of the CD44–HA interplay and especially the role of the arginines important in the recognition of HA. In particular, our work shows why and how R41 is crucial in the recognition of HA in all the three binding modes of which one was detected previously by crystallography [10] and two were observed for the first time in our simulations. Our results expand the molecular-scale knowledge of the CD44–HA interplay and therefore have potential to facilitate the development of novel therapies against conditions such as the metastasis of tumors [32, 33].

## Methods

Table A in S1 File lists the all-atom MD simulations performed in this study. In total, we carried out seven separate simulation sets to evaluate the qualitative and quantitative differences in the formation of CD44-HA binding complexes as well as their characteristics in the microsecond time regime. For complete system descriptions, see section 2 in the Supporting Information (S1 File).

The CD44 residues are numbered according to human CD44-HABD as in the UniProt entry P16070, so that after the cleavage of the N-terminal signal peptide, HABD corresponds to residues 20–169. We also abbreviate the studied HA oligomers as HA_8_, HA_16_, HA_18_, and HA_64_, with the suffix denoting the number of monosaccharides in a given fragment. A particular monosaccharide is referenced by its name, i.e., GlcUA or GlcNAc, followed by the number of the disaccharide unit it belongs to. We designate the disaccharide closest to R41 (the key binding residue) with the number zero, e.g., GlcNAc (0) and GlcUA (0). Then disaccharides in the reducing end of the polysaccharide chain (the GlcNAc terminus) receive a negative order number, e.g., GlcNAc (-1) and GlcUA (-1). Opposite counting is used towards the non-reducing end (the GlcUA terminus), which therefore receives a positive order number. In other words, the numbering of our HA oligomers starts from GlcNAc at the reducing end, the first residue being a GlcNAc (-X), and terminates in GlcUA (+Y) at the non-reducing end of the chain.

### Simulation parameters

We ran the simulations with the GROMACS 4.6.7 simulation software package [34], employing the AMBER99SB-ILDN [35] force field for CD44-HABD and the GLYCAM06h [36] force field for the HA oligomers. These force fields were chosen because they have been shown to capture realistic protein and carbohydrate dynamics and are also compatible with each other [36].

All simulation models used rectangular boxes with periodic boundary conditions. Cubic boxes were used in all the simulations except for the ‘Clustering’ systems, where one dimension of the box was elongated. In these cases, the HA polymer was restrained from its ends (see S1 File for details). All bonds were constrained using the LINCS [37] algorithm, allowing 2.0 fs integration time steps. Electrostatics were treated with the particle-mesh Ewald (PME) [38] method, with a 1.0 nm cut-off distance for the real part. A cut-off of 1.0 nm was applied for van der Waals interactions. Furthermore, we applied long-range dispersion corrections for energy and pressure [39]. Neighbor searching was carried out at every 10 steps. All our systems were simulated in the NpT ensemble. The V-rescale [40] thermostat was used to couple the system to a heat bath of 310 K with a time constant of 0.1 ps, while the Parrinello-Rahman [41] barostat was employed to couple the system to a pressure bath of 1.0 bar, with a time constant of 1.0 ps. At the beginning of each simulation replica, we assigned random initial velocities to the particles from a 310 K Boltzmann distribution. We set the saving rate for trajectories to 1/100 ps. All other non-specified parameters used GROMACS 4.6.7 defaults. To avoid clashes from the construction, before production runs, we minimized the energy of each constructed system with the steepest descent algorithm for 1000 steps and equilibrated the systems for another 2 ps.

## Results

### Hyaluronan can attach to CD44 in three different binding modes

Puzzled by the spread of the observed binding residues in the HA–CD44 interaction, we hypothesize that several binding modes may coexist in CD44-HABD. To this end, we performed two sets of simulations (Table A in S1 File): the ‘Seeding’ simulations (≈6 μs) where we placed the HA close to (1 nm), but not in contact with the binding groove of CD44-HABD; and the ‘Unbound’ simulations (≈10 μs) where we placed the HA far (4 nm) from R41. Therefore, while HA is in principle biased to find the crystallographic binding site in the ‘Seeding’ simulations, it may explore the surface spontaneously without any bias in the ‘Unbound’ simulations.

**Fig 2:**
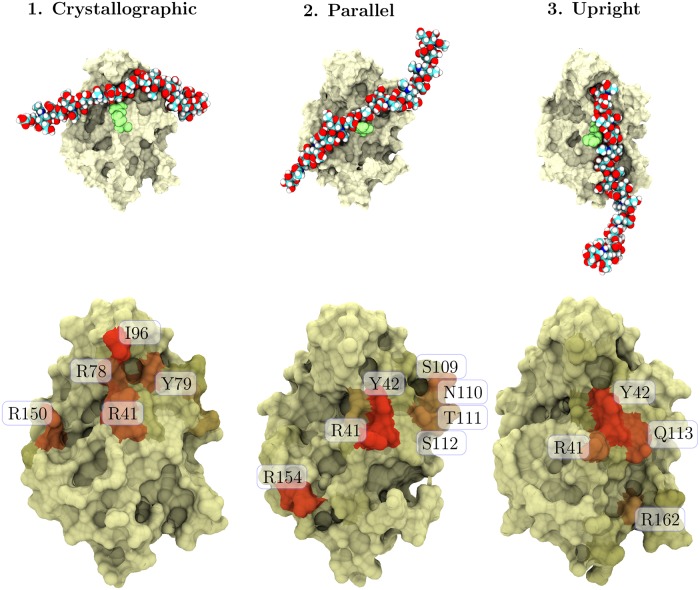
Molecular details of the HA–CD44 binding modes. The three binding modes found in this study. Tan surface represents the CD44 HABD domain, the light green spheres depict R41 (important binding residue), and the multicolored rod is the HA_16_ oligomer. The images shown at the top are snapshots from the starting frames of the gathering simulations (Table A in S1 File). The figures at the bottom describe the most important binding residues in each binding mode. The more reddish the surface becomes, the more contacts it holds, so that red color corresponds to 350 or more contacts on average.

The end structures of all our simulations were found to correspond to three distinct HA–CD44 binding modes. One of these, referred to as the crystallographic mode, has previously been resolved with crystallography [10]. We also observe the upright mode that has been partially proposed in the literature [4], along with a new, so far uncharacterised type of CD44–HA complex that we name the parallel mode (Fig 1). If we orient the CD44 HABD with the N- and C-termini pointing down and R41 pointing towards the viewer, which is the usual orientation in our figures, the crystallographic mode can be recognized from the HA strand as being anchored between the β4–β5 loop (for naming of the secondary structure elements, see Fig A in S1 File) and the tip of the side-chain of R41, located at the α1–β1 motif (Fig 2). This region is also known as the crystallographic binding groove. The HA strand of the parallel complex, on the other hand, lies on top of R41 and in a lower region of the binding groove, lacking contacts with the β4–β5 loop. Both of these binding complexes share the same orientation of the ligand, i.e., the reducing end of HA locates on the right side of the figures. In the parallel mode, however, the HA is tilted counter-clockwise to the viewer (Fig 2), covering a different region of the protein surface. Finally, in the upright complex, HA locates on the right side of R41 and assumes a vertical orientation, with the reducing end of the HA oligomer pointing upwards (Fig 2).

Our findings suggest that HA has considerable interactions with CD44 in three distinct binding modes. This offers a plausible explanation for the wide spatial distribution of the CD44 amino acids critical to HA binding [4, 23]. Furthermore, visualization of all of our simulations showed only a few transitions between the binding modes, and they mostly occurred soon after the binding had initially taken place. These infrequent transitions may reflect failed docking events between HA and CD44 at early stages of the simulations. Hence, we conclude that the binding modes we observed are stable in the microsecond time scales of the simulations.

The free HA ligands in our simulations almost exclusively end up interacting with the crystallographic binding groove or the regions next to it. In the ‘Seeding’ simulations, where the HA_16_ ligand was placed roughly 1 nm from the protein surface to the crystallographic binding groove, the ligand ended up binding with the protein in three out of four replicas in the parallel mode. In the fourth replica, we observed the formation of the crystallographic complex, which later (after 200 ns of simulation) detached spontaneously. However, after 1 μs of simulation, HABD and HA formed the upright complex. Similar binding events were also observed in the five ‘Unbound’ simulations, where the ligand–R41 distance was initially set to 4 nm. In just 400 ns, two replicas formed the parallel binding complex, two formed the upright complex, and one remained less stably bound although still interacting with R41, highlighting the significant role of this crucial binding residue. Overall, the parallel binding mode was observed to be the most frequent in our unbiased simulations, while the crystallographic mode was seen only once in these simulations.

The abundance of the parallel and upright binding modes in the initial stages of the contact suggest that they act as a precursor for stronger binding. It is unclear whether other factors, such as the size, mobility, or orientation of the ligand play any role in determining the binding mode. It is also possible that conformational changes, such as the unfolding of the C-terminal region [12], or post-translational modifications such as N-glycosylation [42] might affect the preference of the available binding modes.

### Three binding modes largely explain the effects of the HA binding residues identified in literature

Table 1 lists the previously identified HA-binding residues of CD44-HABD. To allow comparison with the present simulation results, it also shows the percentage of simulation time these residues were in contact with HA in our simulations for each binding mode.

**Table 1:** HA binding residues of CD44-HABD identified in the literature.

Residue	Crystallogr. (%)	Parallel (%)	Upright (%)	Reference
N25	52.3 ± 0.4	89.7 ± 0.8	0.0	[16, 43]
R29	2.1 ± 0.7	1.1 ± 1.1	11.2 ± 11.2	[4, 8]
K38	0.0	6.9 ± 4.0	73.4 ± 19.6	[4, 8, 23, 25]
G40	9.5 ± 1.5	100.0	39.5 ± 23.6	[4]
R41	100.0	100.0	92.2 ± 4.9	[4, 8, 10, 23, 25]
Y42	100.0	100.0	99.5 ± 0.4	[4, 10, 23]
S43	6.0 ± 6.0	66.1 ± 17.6	98.0 ± 1.3	[4]
I44	0.3 ± 0.3	1.9 ± 1.3	62.5 ± 18.1	[4]
N57	0.0	0.0	20.4 ± 20.4	[43]
K68	0.0	0.0	0.0	[23]
T76	95.1 ± 0.9	2.1 ± 2.1	51.1 ± 17.6	[25]
C77	100.0	33.1 ± 15.5	90.8 ± 2.7	[10, 25]
R78	100.0	100.0	92.8 ± 3.3	[4, 10, 23, 25]
Y79	100.0	85.2 ± 8.2	93.4 ± 3.2	[4, 10, 23, 25]
G80	0.1 ± 0.1	0.0	0.1 ± 0.1	[25]
I88	100.0	10.3 ± 3.4	78.7 ± 9.0	[10]
N94	77.2 ± 2.5	2.5 ± 2.5	26.5 ± 7.9	[10]
I96	100.0	16.5 ± 7.5	96.9 ± 0.8	[10, 25]
C97	100.0	2.9 ± 2.9	93.1 ± 1.2	[10, 25]
A98	100.0	41.9 ± 15.3	89.4 ± 4.7	[10, 25]
A99	100.0	10.2 ± 4.6	73.7 ± 19.3	[10, 25]
N100	13.2 ± 2.5	1.5 ± 1.2	30.7 ± 12.8	[23, 43]
H101/N101	47.8 ± 10.7	1.6 ± 0.0	30.3 ± 14.6	[10, 25]
Y105	89.6 ± 5.6	46.8 ± 15.1	36.3 ± 14.6	[10, 23, 25]
I106	35.3 ± 13.7	18.9 ± 10.2	0.5 ± 0.5	[25]
L107	99.9 ± 0.1	91.8 ± 5.5	63.9 ± 24.2	[25]
N110	92.1 ± 5.7	100.0	13.6 ± 7.4	[23, 43]
D115	7.8 ± 2.7	93.9 ± 3.0	20.7 ± 18.4	[25]
N120	0.0	0.0	17.5 ± 17.5	[16, 43]
N149	37.1 ± 4.5	36.5 ± 20.3	0.0	[25]
R150	88.0 ± 2.7	37.0 ± 28.5	0.0	[4, 8, 14]
D151	68.1 ± 7.8	37.4 ± 26.2	0.0	[25]
G152	61.9 ± 8.6	65.2 ± 20.3	0.0	[25]
R154	3.2 ± 0.5	81.0 ± 13.7	0.1 ± 0.1	[4, 8, 14]
Y155	1.2 ± 0.9	44.2 ± 22.1	0.0	[4]
K158	0.0	17.0 ± 11.9	35.9 ± 18.2	[8, 14]
R162	0.8 ± 0.4	0.0	98.1 ± 1.0	[4, 8, 14]
N164	10.7 ± 7.6	0.3 ± 0.1	68.1 ± 18.8	[4]
E166	16.0 ± 9.3	0.4 ± 0.4	72.4 ± 19.1	[4]

The first column gives the HABD residue. The second, third, and fourth columns provide the binding percentage in each binding mode. This figure is calculated as the number of frames where the given residue is closer to 0.6 nm of HA, and then dividing it with the total number of simulation frames for each replica in each binding mode. The errors are standard errors. The last column lists the studies that have identified the residue in question as important for HA binding.

The Banerji et al. study found 13 residues of HABD to make prominent contacts with the bound HA: R41, Y42, C77, R78, Y79, I88, N94, I96, C97, A98, A99, H101 (N101 in human sequence), and Y105 [10]. These residues are exclusively located in the crystallographic binding groove and form a coherent surface under the bound ligand. Our data from the crystallographic binding mode correlate well with these residues (see Table 1), excluding N94 and H101 (N101 in human CD44) which are both located at the β4–β5 loop. The reason for these minor differences most likely lies in the slightly different folding of this loop in the murine structure (PDB:2JCQ) determined by Banerji et al. [10] and the human protein (PDB:1UUH) [4] used in the present study.

Earlier, Peach et al. discovered two clusters of binding residues, one in the link module and another in the C-terminal flanking regions [8]. The former comprises residues R29, K38, and R41. While R41 was clearly demonstrated through alanine mutation to be crucial for HA binding, both R29 and K38 also shared a moderate effect on the recognition. Residue K38 lies close to both the binding groove and the C-terminus. In our simulations, this residue realizes contacts with HA exclusively in the upright mode (79.3% of the aggregate simulation time, see Table 1). On the other hand, R29 maps to the other face of the protein and is not observed to establish contacts with the bound HA in any of our simulations. The second binding cluster that Peach et al. discovered is located in the C-terminal extension of the HABD. It is composed of residues R150, R154, K158, and R162. The mutation of these residues to alanine results in a moderate decrease in HA binding. However, compound mutations, such as the K158A/R162A double mutant, caused a much more notable decrease. In our simulations of the crystallographic mode, R150 interacts with HA in 88% of the frames. In the parallel mode, R154 binds to HA in 80.8% of the frames. Finally, in the upright mode, R162 is one of the primary binding residues interacting with HA in 98.2% of the simulation frames. In their crystal structure, Banerji et al. [10] also observed R150 residue to bind to HA transiently but attributed a quite small role for these transient interactions. In light of our simulation data, these flanking residues seem to be important, although each is contributing to a different binding mode. This could explain why Peach et al. found these basic residues to cause a noticeable decrease in HA binding only as compound mutations.

Later, Bajorath et al. employed the first *in silico* model of CD44-HABD to select potential residues for a mutation assay [23]. As a result, nine residues were found to be significant for HA recognition: K38, R41, Y42, K68, R78, Y79, N100, N101, and Y105. The majority of these residues are in line with the Banerji et al. study [10]. For instance, Bajorath et al. found residues R41, Y42, R78, and Y79 at the center of the crystallographic binding groove to be vital for HA binding [23]. Further, supporting their role, these residues are also highly connected to HA in all of the three binding modes studied here, see Table 1. Additionally, K38 was identified as important for HA binding, agreeing well with the findings of the Peach et al. study. Only the role of K68 was left elusive, as it maps to the other side of the HABD as the binding groove or the ordered C-terminus [10]. It was not found to bind HA in any of our systems either. Given its proximity to R29 that was identified in the Peach et al. study, it is plausible that these residues are linked to some other affinity-modifying mechanism, such as the aggregation of receptors.

In addition to the mutation assays, NMR has been used to identify HA binding residues in human CD44 HABD. For example, Takeda et al. identified residues in the binding groove (T76, C77, R78, Y79, G80, I96, C97, A98, A99) to be masked by the presence of the ligand, implying that the ligand might have been bound in the crystallographic manner [25]. However, they also noted large chemical shift changes upon HA binding in residues R41 and K38, from which the latter is not involved in the crystallographic binding. This could imply that other binding modes, such as the upright mode, were present in their HA–HABD constructs. Furthermore, Takeda et al. observed large chemical shift changes in the C-terminal region of HABD (β8 and β9 sheets) [11, 25]. They attributed these changes to the partial disordering of the C-terminus. Teriete et al. observed somewhat a similar rearrangement at the flanking regions of HABD [4]. They also evaluated HA binding residues in HABD based on their chemical shifts. Residues K38, G40–I44, R154–Y155, and N164/E166 gave the most prominent signals, while noticeable changes were also observed with residues R78, Y79, R150, R29, and R162, thereby largely agreeing with our observations and with either the Peach et al. or the Bajorath et al. study.

The unfolding of the C-terminus from the O to the PD conformation can also explain why the basic C-terminal residues were found to be important in HA binding in the above studies, most notably in the Peach et al. study. As Favreau et al. showed, the C-terminus gains considerable conformational flexibility in the PD conformation, allowing the basic residues to readily come into contact with the bound HA ligand [14]. Hence, this can explain why Ogino et al. observed CD44 population to favor the PD conformation in the ligand bound state [12]. However, regardless of the changes in the C-terminus, several of the above studies have listed K38 as an important binding residue even though it is not located in the binding groove or in the unfolding C-terminal extension. This finding combined with our observation that K38 is important only in the upright mode could imply that there exists additional HA–CD44 binding modes outside the crystallographic one.

There are five N-glycosylation sites in CD44-HABD (N25, N57, N100, N110, and N120) [4], so the presence or absence of N-glycans might also alter the binding. Furthermore, English et al. have shown that all the five glycosylation sites on HABD host N-glycans when expressed with cancer cells [16]. Several studies have probed the effect of mutating these N-glycosylation sites on HA binding [15–17, 43]. For instance, mutating N25 or N120 to serine increased HA binding considerably, whereas mutating the other three sites displayed only a negligible effect [16]. On the other hand, Bartolazzi et al. showed that mutation of any of the five N-glycosylation sites is enough to abolish HA binding [43]. The contradictory shreds of evidence might be explained by, for example, cell-type specific differences. However, due to these discrepancies, the role of the N-glycosylation site residues for HA binding remains unclear. In our simulations, the non-glycosylated N25 and N110 were both observed to be in contact with HA over a half of the simulation time in every binding mode, while the rest of the N-glycosylation sites made fewer contacts with HA. Considering that Peach et al. and Bajorath et al. found residues (R29 and K68) close to N120 and N57, from which N120 was deemed important in the English et al. study, it is possible that the existence of N-glycans gives rise to or amplifies some binding modes. It is also worthwhile to notice that the studies listed in Table 1 use different expression platforms for their CD44-HABD constructs, which might differ considerably in their glycosylation content, thereby affecting the results obtained.

All in all, only five residues in Table 1 (R29, N57, K68, G80, N120) lack any HA-interaction over 30% in our simulations. These residues map to the “back” side of HABD. All the other residues, locating at the “front” face (i.e., the face of the binding groove) of HABD have over 30% contact time with the ligand in at least one binding mode. Hence, the existence of multiple binding modes seems a plausible explanation for the observed spread of the HA-binding residues.

### Comparison between the binding modes reveals that they all are dependent on interactions with selected key residues and also share many common features

Our simulations showed that three different binding modes establish spontaneously between HA and CD44. To further characterize the structural and dynamical features of these binding modes, we performed the ‘Gathering’ simulations, where each simulation was prepared in a well-defined binding mode: crystallographic, parallel, or upright (Fig 2). In total, three independent replicas for each binding mode were simulated for 1 μs. The binding mode remained unchanged in every one of them. For detailed descriptions of the structural features of each binding mode, see section 3 in S1 File.

**Fig 3:**
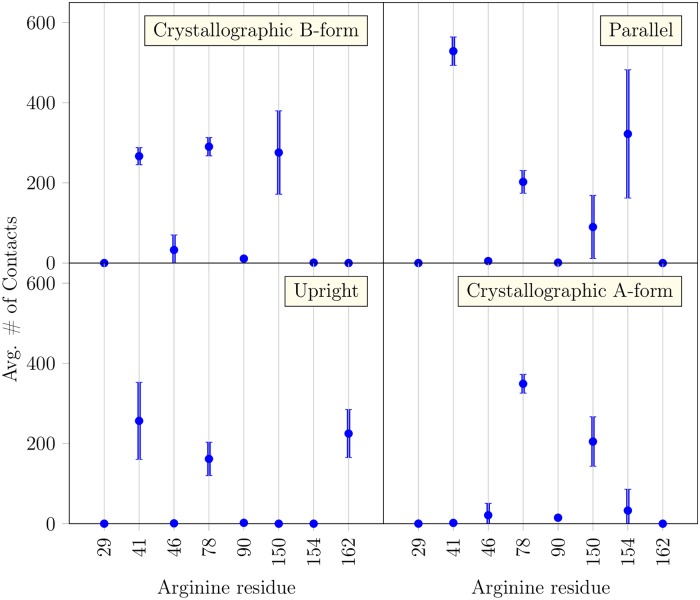
Contacts between HA and the arginines of CD44. Average number of contacts of HA with each arginine residue in the CD44 HABD, calculated from the three replica systems (3 × 1000 ns) per each binding mode and the A-form simulations spanning 300 ns (Gathering simulations in Table A in S1 File). The error bars represent standard errors.

**Fig 4:**
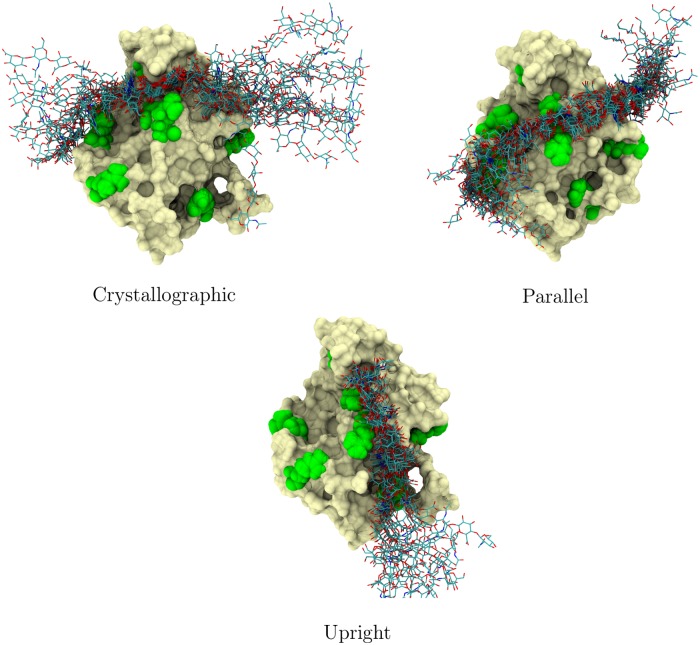
Stability of each binding mode. Fluctuations observed in the HA structure for each binding mode. The tan surface represents the protein in the first frame of the trajectory. The green spheres are the arginine residues. The multicolored sticks represent the HA conformation, plotted here every 50 nanoseconds of the trajectory. The frames have been taken from the first replica system of each Gathering simulation (Table A in S1 File) (data based on the other replicas were consistent with the data shown here).

In Fig 3, we depict for each binding mode the contacts between HA and every arginine residue of CD44. They display a unique contact signature or fingerprint, which correlates well with the regions of lesser HA mobility in Fig 4. Hyaluronan-binding proteins usually contain arginine-rich motifs [2] and their mutations often substantially influence the hyaluronan binding affinity [8]. In the case of CD44, these arginines are very widespread on the surface of the protein. One possible explanation for this counter-intuitive observation is the existence of several binding modes.

Despite the observed clear differences between the binding modes (see also Figs D to O in S1 File), they also share common features. To begin with, all binding modes share a strong interaction with R41 and the neighboring Y42. Without exceptions the contact region around R41 and Y42 is the least mobile of all the HA–HABD contacts, denoting clear binding stabilization, see Fig 4. This stabilization illustrates why R41 mutations lead to a considerable decrease in the binding affinity despite the observation that the CD44-HABD 3D structure seems unaffected in complementary antibody assays [8]; any change around R41 would perturb all three binding modes. Moreover, the fact that this modification strongly inactivates HA binding suggests that the three binding modes found in this work may be the only significant ones existing [8]. Another important feature shared by all the binding modes is the extended hydrogen bond network region towards the reducing end of the bound HA, between T108 and Y114. Finally, the interaction with R78 is also shared by all three modes, while it seems to be particularly prominent only in the crystallographic and upright modes.

While all binding modes share features that can be considered to be the core of their binding in the CD44-HA complexes, our results show that each mode also has other interactions that partially stabilize their structure. In each binding mode, our data show that HA establishes hydrogen bonds with at least another basic residue placed between 3–4 nm towards the non-reducing end (see Fig I in S1 File): R150 for crystallographic; R154 and partially R150 for the parallel; R162 for the upright mode. This common feature seems to play an important role in stabilizing the mobile end of the non-reducing HA end, as can be seen in Fig 4. These two flanking arginines R41-RX (where X depends on the binding mode) anchor the HA oligomer to the surface of CD44-HABD with 6 to 8 monosaccharides, thereby increasing the stability of binding. This provides a reasonable explanation to why the binding is severely diminished with very short fragments of HA, e.g., HA_4_ [4]. There is conflicting experimental evidence regarding the stabilization by the flanking arginines. While Peach et al. found decreased binding upon mutation (R150A, R154A, R162A) consistent with our results [8], Banerji et al. found that the same mutations have no effect on HA binding in a HA-ligand binding assay [10]. The source of this contradiction is currently unknown but our data align with the observations of the former study.

It is possible that the parallel and upright binding modes represent metastable states, as described in Ref. [44], preceding the crystallographic complex. Supporting this, the upright and especially the parallel mode are relatively abundant in the timescales of our simulations, while the crystallographic form is seen to form only twice. On the other hand, we did not observe any direct transitions between any of the binding modes. Given our approximately 50 microseconds of sampling, this implies that such transitions are rare in the timescales of simulations. Taking into consideration that the time scales probed here are short compared to the biologically relevant ones, we proceeded to study the free energy profile of each binding mode to quantitatively assess their importance.

### The crystallographic mode has the lowest free energy but the other two modes are also accessible

**Fig 5:**
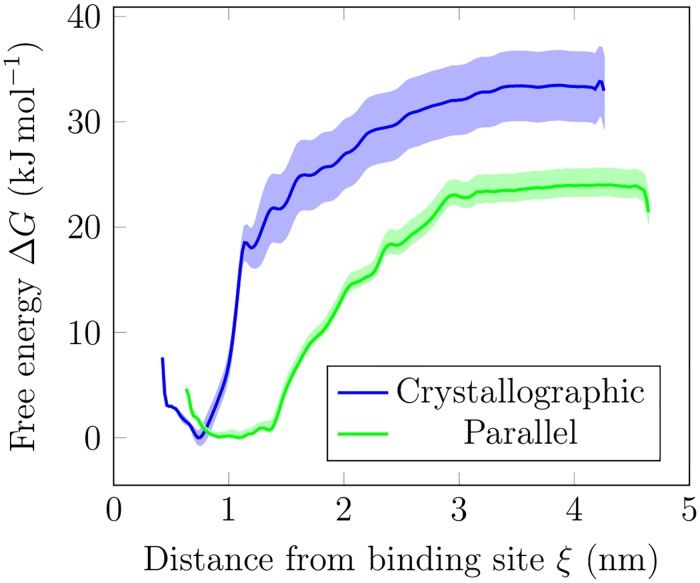
CD44–HA free energy profiles. HA–CD44 free energy Δ*G* (kJ mol^−1^) as a function of distance *ξ* to the binding site. Pull groups are the centre of mass of HA and the centre of mass of the HABD residues 75–79. The error bars, based on weighted histogram analysis, are shaded accordingly.

We computed the free energy profiles of detaching the HA from the HABD surface with the umbrella sampling technique. We investigated the binding free energy of HA_8_–HABD in two cases: the parallel complex and the crystallographic complex, see Fig 5. For the parallel form, we obtained a free energy difference of −22 kJ mol^−1^ (∼8.8 k_B_T), while the crystallographic mode indicated a clearly stronger value of −33 kJ mol^−1^ (∼13.2 k_B_T). The orders of magnitude of these values are in agreement with experimental observations, where attachment of HA oligomers of this size to CD44-HABD has been found to be reversible [18]. Our estimates for the binding strength also correlate well with the experimentally observed values [10, 18]. The free energy difference between the two binding modes (∼4.4 k_B_T) suggests that the crystallographic mode is 80 times more favorable than the parallel mode, although the latter is more probable in our simulations. If we use the ratio of the integrated populations as obtained from free energy profiles to evaluate the probability [45] of each mode, the difference reduces to ∼20-fold. This implies that the entropic component of the free energy favors the parallel mode over the crystallographic. In either case, the difference of free energies is small enough for both modes to coexist to a substantial degree. The parallel mode might become a plausible alternative especially in a scenario, where the availability of the crystallographic binding site is hindered, e.g., due to N-glycosylation.

Calculating the free energy with the umbrella method has its caveats, see S1 File. Being aware of such limitations, we decided to support our free energy calculations with an alternative measurement method. Inspired by the electrophoresis experiments used to determine the strength of interaction between HA and CD44-HABD [17], we designed an *in silico* counterpart.

Taking advantage of the total negative charge of HA, we applied an external electric field to the HA–CD44-HABD complex. In our simulation experiments, the CD44-HABD was fixed and oriented so that the HA binding groove was facing the *in silico* equivalent of the positive electrode (anode). The primary advantage of these simulations is the more natural detachment of the ligand compared to the umbrella free energy simulations, thereby mimicking this biologically relevant process in a more appropriate setting. In practice, we applied two fields with different strengths. In the first set, ‘E-field strong’, the field was strong enough to ensure that detachments took place in short simulation times (up to 20 ns), see Table 2. We then performed a total of 20 simulations for each binding mode and calculated the number of contacts between the HA ligand and CD44. When the number of contacts between HA and HABD reached zero for the first time, we considered the ligand as “detached”.

**Table 2:** In silico electrophoresis as qualitative measurement of stability.

System (binding mode)	Detachments	Average detachment time (ns)
Crystallographic B-form	5%	8.08 ± N/A
Parallel	95%	4.89 ± 2.38
Upright	20%	14.09 ± 3.76
Crystallographic A-form	25%	7.63 ± 5.35
Crystallographic B-form	0%	0 ± N/A
Parallel	20%	77.64 ± 67.33
Upright	5%	91.50 ± N/A

Relative number of ligand detachments for each binding mode and their average detachment times for the ‘E-field strong’ (upper panel, 20 ns simulations) and ‘E-field weak’ (lower panel, 200 ns simulations) simulation sets, respectively. Data are averaged from 20 simulation trajectories per system, where only the “detached” trajectories are considered when calculating the detachment times. N/A stands for not available (due to limited amount of data).

The results (Table 2) obtained agree with our previous calculations. The crystallographic mode seems to be the strongest again, while the parallel mode seems to be the weakest. The upright mode shows intermediate behavior. Interestingly, our data also show that the crystallographic A-form is weaker than its B-form counterpart, displaying a strength similar to the upright mode. This result supports the view that the A-form is just an intermediate state to the B-form, which is further stabilized by hydrogen bonds with R41 [14]. It is important to stress that the strong field applied in this set is certainly unphysical and results in substantial water orientation, minimizing its dipole moment, see Fig C in S1 File. Naturally, this changes the solvation environment of the protein and HA, significantly affecting the binding affinity we aim to measure.

Due to the field-induced effects, such as the water orientation, we performed a second simulation set, ‘E-field weak’, where we reduced the strength of the field to a tenth of its original value. In this set, the orientation of water was significantly closer to the orientation of bulk water in zero-field (see Fig C in S1 File), and therefore the environment of the HA–CD44-HABD complex is here appropriate compared to biological conditions. Due to the decrease in the strength of the field, each simulation was extended to 500 ns, totaling 10 μs of simulation per binding mode.

The results with the weaker field (Table 2) are consistent with the other two methods, indicating that our estimates for the binding strength are reliable and robust. Additionally, our ‘E-field weak’ simulations provide interesting information about the intrinsic stiffness of each binding mode. As seen in Fig B in S1 File, where we plot the distance of HA (the methyl group of GlcNAc(0)) to R78 during the detachment simulations, each binding mode displays a unique signature. While the effect of the electric field is not noticeable in the crystallographic complex, the upright complex fluctuates heavily around its initial equilibrium position. We also observed a few events were there is partial detachment. In these cases, the HA strand, however, remained bound through interactions with the residues 108–114 of the protein. This points to the existence of regions which can stabilize or initiate a given binding mode. Finally, we observed that in the parallel mode there is a clear rearrangement or detachment when one applies the field. It is important to mention that in this mode the R78 interaction was not very relevant even though it was used as a reference point in the protein when calculating the distances.

We conclude that the sampled binding modes have different free energy profiles. The crystallographic mode is the most favorable one, while the parallel mode is the least favorable. However, we also see that the weaker binding modes, i.e., parallel and upright, have significantly larger regions of interaction with CD44-HABD, thereby increasing the probability of their occurrence. This is largely the reason why we observed a considerable variation in the free energy when calculated by the difference between the bulk and the minimum in the free energy profile or by integrating the profile independently for the bound and unbound states. Therefore, our results suggest that while the crystallographic mode plays a crucial role in the HA–CD44-HABD interaction, the metastable modes also increase the HA–CD44-HABD binding constant (sum of the binding constants of each binding mode). Furthermore, in real biological systems, one rarely maintains pure equilibrium conditions. The orientation of the molecules, blood flow, or interaction with other molecules might induce an external bias to CD44–HA complexes resting on the plasma membrane and biasing the preferred binding mode.

### The A-form of the crystallographic binding complex is an intermediate state in the formation of the B-form complex

The crystallographic binding complex can be found in two conformational states, the so-called A- and B-forms, see Fig P in S1 File. While the HA ligand may be bound to both conformations, the A-form lacks direct R41–HA contacts, whereas the B-form enables the R41 side-chain to form two direct hydrogen bonds with the bound ligand [10]. Another computational survey recently discovered the molecular basis for the conformations: the ϕ dihedral in Y42 backbone acts as a bistable switch, altering the shape of the β1–α1 loop region, which in turn causes the R41 side-chain to flip between these two conformations [13]. Furthermore, many studies suggest that the presence of HA in the binding groove stabilizes the B-form [10, 13, 14].

We found the spontaneous formation of the crystallographic B-form complex in two occasions, see supplementary video. Importantly, these events occurred in equilibrium simulations, meaning that they were not initiated by external perturbations. Instead of binding directly to the crystallographic B-form structure, we found on both occasions that HA binds first to the A-form structure. The B-form complex formed only when HA had aligned correctly to the binding groove, a process most distinctively characterized by the methyl group of GlcNAc(0) pointing to the hydrophobic pocket. Given these data, the formation of the crystallographic complex is described by the following equation:

$$\begin{array}{c} \text{CD}44^{\text{A}-\text{form}}+\text{HA }⇋\text{ CD}44^{\text{A}-\text{form}}{\text{HA}}^{\text{crystallographic }}⟶\text{ CD}44^{\text{B}-\text{form}}{\text{HA}}^{\text{crystallographic}} \end{array}$$

This finding is in line with the current predictions and implies that the crystallographic A-form complex is an intermediate state in the formation of the B-form complex. Providing further support for this notion, we did not observe spontaneous B-to-A flips in the HA-bound systems once the B-form had established. We have, however, recorded such transitions in ligand-free simulations or in cases where the ligand has detached (see Fig Q in S1 File). This observation is in line with previous computational studies that revealed the type-B conformation to be the favorable conformation in the ligand bound HABD [14].

In terms of the interactions with the ligand, the differences between the A and B complexes are subtle. In fact, the lack of contacts with the R41 side-chain is the only notable difference between the two conformations (Fig 3). As already shown by Favreau et al. in a previous computational study, the flipping of the R41 side-chain suffices to provide additional stabilization for the complex. This is supported by the data in Table 2, which show that the A-form complex dissociates more often under the electric field than the B-form complex. Finally, it is important to stress that the A and B conformations are only relevant with the crystallographic binding mode. In the parallel mode, for instance, the transition cannot happen as HA lies on top of R41, restricting its movement.

### HA and CD44 slide along each other, leading to spatially restricted motion that can foster the aggregation of CD44

HA exists in a variety of sizes. Its biological effects depend on its molecular weight [46]. Low molecular weight HA (i.e., up to 20 carbohydrate units) has, for example, been implicated in the stimulation of cell proliferation [47], while high molecular weight HA inhibits the same process [48]. Interestingly, the length of HA has been reported to influence also the aggregation of the CD44 receptors. In a recent study, polymeric HA stimulated the aggregation of CD44 *in vivo* in multiple cell types [21]. Oligomeric HA comprised of 6–20 carbohydrate units, on the other hand, disrupted this aggregation effect. In another study, increasing the length of HA was observed to augment the CD44–HA binding *in vitro*, with a saturation point at molecular weights of 262 kDa, corresponding to polymers of almost 700 disaccharide units [18]. Provided that the HA polymers are long enough, incrementing the surface density of CD44-HABDs seems to result in the same effect [18]. Finally, the aggregation affinity depends on the CD44 variant, suggesting that differences in the HA–CD44 interaction also play a role [19].

In light of the above, we elucidated the molecular details of the interaction between a high weight HA polymer and multiple CD44s. In practice, we conducted two replica simulations termed “Clustering”, where we simulated two CD44-HABD proteins together with HA_64_, spanning the length of the simulation box (30 nm × 8 nm × 8 nm, see Methods in section 2.3 in S1 File for details). Both HA and CD44-HABD are restrained, however they able to move freely along the long edge of the simulation box (i.e., in the direction parallel to the HA polymer). Overall, the conditions simulated in this system mimic those present at a cell membrane, with the CD44 receptors protruding from the plasma membrane and interacting with a rod-like HA polymer, like in the pericellular matrix of a cell [46, 49].

**Fig 6:**
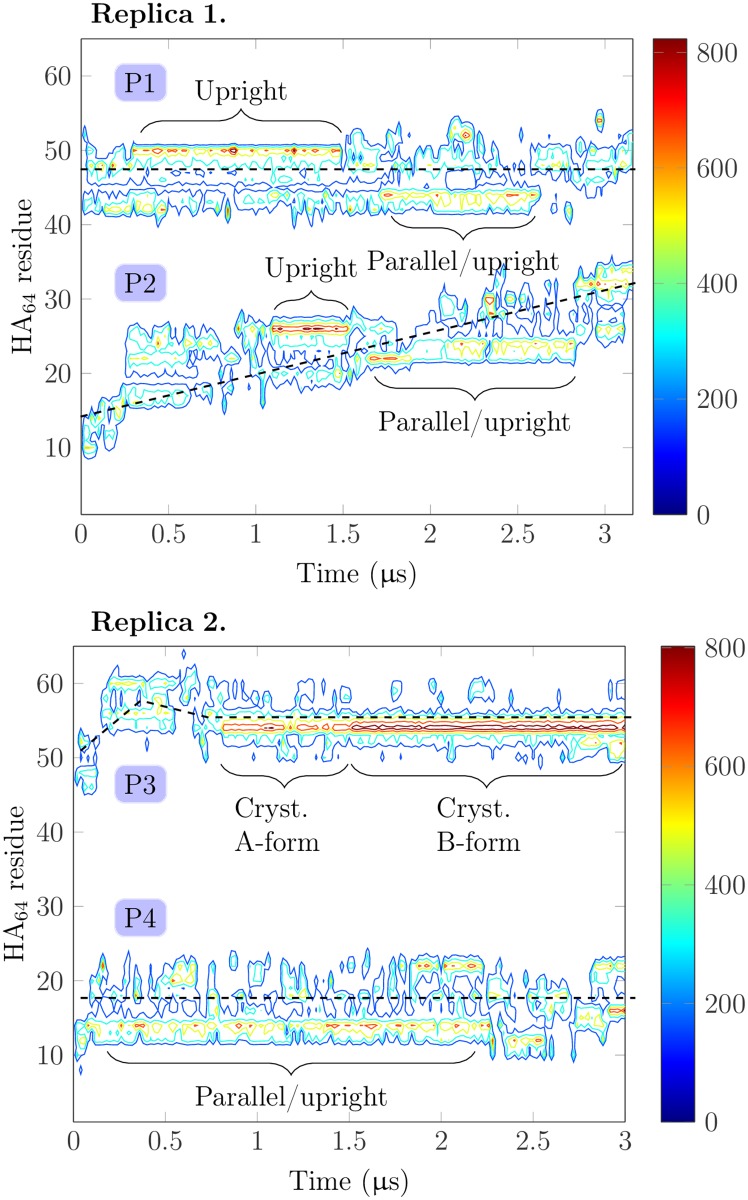
Time-evolution of CD44–HA interaction. Contour plots describing the contacts of HA residues with two CD44 proteins as they diffuse along HA. Data are shown for two independent replica systems (proteins P1 and P2 in replica 1, and proteins P3 and P4 in replica 2). Vertical axis runs through the HA residues, horizontal axis depicts time through the 3 *μ*s simulation time span, and the contours highlight the number of HA-CD44 contacts in terms of color code shown on the right. The dashed lines guide the eye to visualize the motion of the proteins. The data are calculated from the ‘Clustering’ simulations in Table A in S1 File.

CD44 proteins can move along the HA_64_ strand. While the first protein (P1) in the first replica simulation remained stationary relative to HA, the second protein (P2) moved a stretch of roughly 20 carbohydrate units along the HA_64_ strand during the 3 *μ*s simulation (see the upper graph in Fig 6). This observation illustrates that HA can effectively capture CD44 HABDs resulting in an increasing local CD44 concentration along HA that can foster aggregation. Additionally, HA limits CD44 diffusion into one dimension (along the HA polymer). This dimensional restriction influences the effective contact times and relative orientation of the CD44–CD44 pairs, which can further enhance the aggregation kinetics of these receptors [50]. The diffusion of the protein is possible due to periodic, yet transient (10–20 ns), partial detachments from HA. In this replica, neither of the proteins remained in any specific binding mode but kept sampling both the parallel and upright modes, the latter being possible due to the tilting of the protein. The most well-defined binding process occurred with the diffusing protein (P2) at 1.1 *μ*s (see Fig 6 (top)), when the upright complex was formed. However, it lasted only about 400 ns before another detachment. Furthermore, in this simulation, both proteins always interacted spontaneously with HA_64_ with their R41-containing face, again highlighting its importance in recognition.

In the second replica simulation, the protein P3 formed a crystallographic binding complex (see S1 Video). After the initial contact to residues 54–62 of the HA_64_ polymer, the protein moved a stretch of three carbohydrate units before binding HA in the crystallographic mode. At this stage, the protein was in the type A conformation, but after roughly 700 ns it turned into the type B form, thereby completing the binding. The formation of the A-form crystallographic complex and the commencing of the type B conformation can both be observed as increases in the contacts in the lower graph of Fig 6. These occur at 750 and 1500 ns in the trajectory, respectively. Importantly, these binding events completely halted the diffusion of the protein relative to HA_64_, highlighting the strength of the crystallographic binding complex. The protein P4, on the other hand, was loosely bound throughout the simulation, sampling both parallel and upright binding modes, analogously to the second protein in the first replica.

The above observations provide a plausible explanation for the existence of multiple binding modes with varying affinities. With only the strong crystallographic B-form, the relative movement along the HA polymer would not be possible, and therefore the aggregation of the CD44 receptors (in terms of kinetics) along HA might not be enhanced. Supported by the fact that the parallel and upright modes are present in microsecond time scales, our simulation data support the view that a non-zero fraction of the CD44 population is bound to HAs through these metastable binding modes, thereby fostering their diffusion along HA.

## Discussion

Based on an extensive analysis of microseconds of atomistic MD simulation data generated in this study, there are three different binding modes for the CD44–HA interaction. We call them here the ‘crystallographic’, ‘parallel’, and ‘upright’ modes. From these mutually exclusive binding orientations, the crystallographic mode is well characterized in the existing literature, while the latter two were observed for the first time in the present study. The fact that we observed these binding modes in this work stems from the system set-up, which allows the components (i.e., CD44 and HA) to move freely in solution.

The spontaneous formation of these three binding modes in our unbiased simulations gives an explanation as to why the previous mutagenesis and NMR shift studies [4, 8, 23] identified so many topographically widespread CD44 residues to be involved in the recognition of HA. The other possible explanation given in the literature is the partial disordering of the C-terminal extension of HABD. However, there is no obvious reason why these two explanations would rule each other out.

Our estimates for the relative binding affinity further revealed that the crystallographic mode, first described in Banerji et al. in 2007, is the strongest of the three modes. Meanwhile, the parallel mode is the weakest of the three but also the most frequently found orientation in our simulations. However, the differences in binding affinities are quite small and within a range of up to 5 k_B_T, implying that all the modes can coexist at the same time with non-zero proportions, thus increasing the binding constant. Especially the existence of N-glycans on HABD might readily alter the relative propensity of these binding modes.

Based on our work, every arginine residue on the same face of the protein as R41 seems to be useful in stabilizing some of the characterized binding modes. However, R41 is the only residue interacting with every mode, highlighting its importance in the recognition. R78 also participates in all of the modes, however, its contribution to the parallel mode is minimal. Furthermore, R150, R154, and R162 are all important for at least one of the binding modes. This contrasts with the results of Banerji et al. who only considered a static view based on the crystallographic binding mode found by X-ray. However, the importance of these flanking arginines might explain why short (1–2 disaccharide units) HA fragments do not bind strongly to CD44. Finally, the fact that R41A mutation abolishes HA binding suggests that the three R41-dependent binding modes found in this work are the most significant ones.

We also further clarified the structural details of the well-characterized crystallographic binding mode. Namely, this is the first study where the spontaneous formation of this binding complex has been recorded, confirming that the HA oligomer first binds to CD44 in the A-form (‘open’ conformation) in a crystallographic manner, after which the B-form (‘closed’ conformation) commences. Upon the process of attachment, CD44 was also observed to diffuse along the HA strand. This suggests that HA can restrict the diffusion of CD44 proteins to one dimension (to take place along the HA polymer) which together with the larger local CD44 concentration along HA can potentially promote aggregation (kinetics) of these membrane-bound receptors. Our data also suggest that the crystallographic binding complex alone is too strong for the diffusion to take place, thereby providing an additional reason for the existence of the weaker modes.

Overall, aggregation is a viable regulation mechanism for membrane proteins. It can, for example, exclude proteins that are aggregated from transmitting signals across the membrane [3]. Although many details of CD44-mediate signaling are still unclear, it could be regulated similarly.

Lastly, there is reason to keep in mind that CD44 is (in its natural form) a glycoprotein housing five possible N-glycosylation sites in the HABD. It is possible or even likely that the presence of N-glycans can switch the population of proteins to favor some HA binding mode over the others, or even generate totally novel interaction ways for these complex macromolecules. Hence, it is also possible that the lack of N-glycans leads HA and CD44 to interact in ways that are less relevant in mammalian cells.

## Supporting information

S1 File Supplementary information file. The file includes a thorough description of the simulated systems and methods, and a more detailed description of the Hyaluronan–CD44 binding modes.
(PDF)

S1 Video Supplementary information video. Visualization of one of the Clustering simulations, showing the full chain of events that lead to spontaneous formation of the crystallographic binding mode.
(MP4)
