# Supplementary material for: Atomistic fingerprint of hyaluronan–CD44 binding
Source: PLoS Comput Biol. 2017 Jul 17;13(7):e1005663. doi: 10.1371/journal.pcbi.1005663 (PMC5549728; doi:10.1371/journal.pcbi.1005663)
Supplement: S1 File — The file includes a thorough description of the simulated systems and methods, and a more detailed description of the Hyaluronan–CD44 binding modes. (PDF) [file pcbi.1005663.s001.pdf]

# Supporting information for: Atomistic fingerprint of hyaluronan–CD44 binding

Joni Vuorio,[†,‡] Ilpo Vattulainen,[†,‡,¶] and Hector Martinez-Seara[*,§,†]

†*Department of Physics, Tampere University of Technology, Tampere, Finland*

‡*Department of Physics, University of Helsinki, Helsinki, Finland*

¶*MEMPHYS - Centre for Biomembrane Physics, University of Southern Denmark, Odense, Denmark*

§*Institute of Organic Chemistry and Biochemistry, Academy of Sciences of the Czech Republic, Prague, Czech Republic*

E-mail: hseara@gmail.com

# Contents

**1 Structure of the CD44 hyaluronan binding domain** **S2**

**2 Methods** **S4**

2.1 Descriptions of systems and simulations . . . . . . . . . . . . . . . . . . . . . . S4

2.2 System construction . . . . . . . . . . . . . . . . . . . . . . . . . . . . . . S6

2.3 Parameters for systems with two CD44 HABDs and one HA polymer . . . . S7

2.4 Special parameters for biased simulations . . . . . . . . . . . . . . . . . . . . . . S8

2.5 Analysis details . . . . . . . . . . . . . . . . . . . . . . . . . . . . . . . . . S11

**3 Detailed description of Hyaluronan–CD44 binding modes** **S12**

3.1 Crystallographic mode . . . . . . . . . . . . . . . . . . . . . . . . . . . . . S13

3.2 Parallel mode . . . . . . . . . . . . . . . . . . . . . . . . . . . . . . . . . . S22

3.3 Upright mode . . . . . . . . . . . . . . . . . . . . . . . . . . . . . . . . . . S26

**4 A-to-B transitions in the crystallographic binding mode** **S29**

**5 Appendix 1: table of contents for each binding mode** **S31**

**References** **S36**

# 1 Structure of the CD44 hyaluronan binding domain

Figure A shows a cartoon representation of the human CD44 Hyaluronan Binding Domain (HABD) together with the naming convention for the secondary structure elements used in this work, following the convention in Ref.[S1].

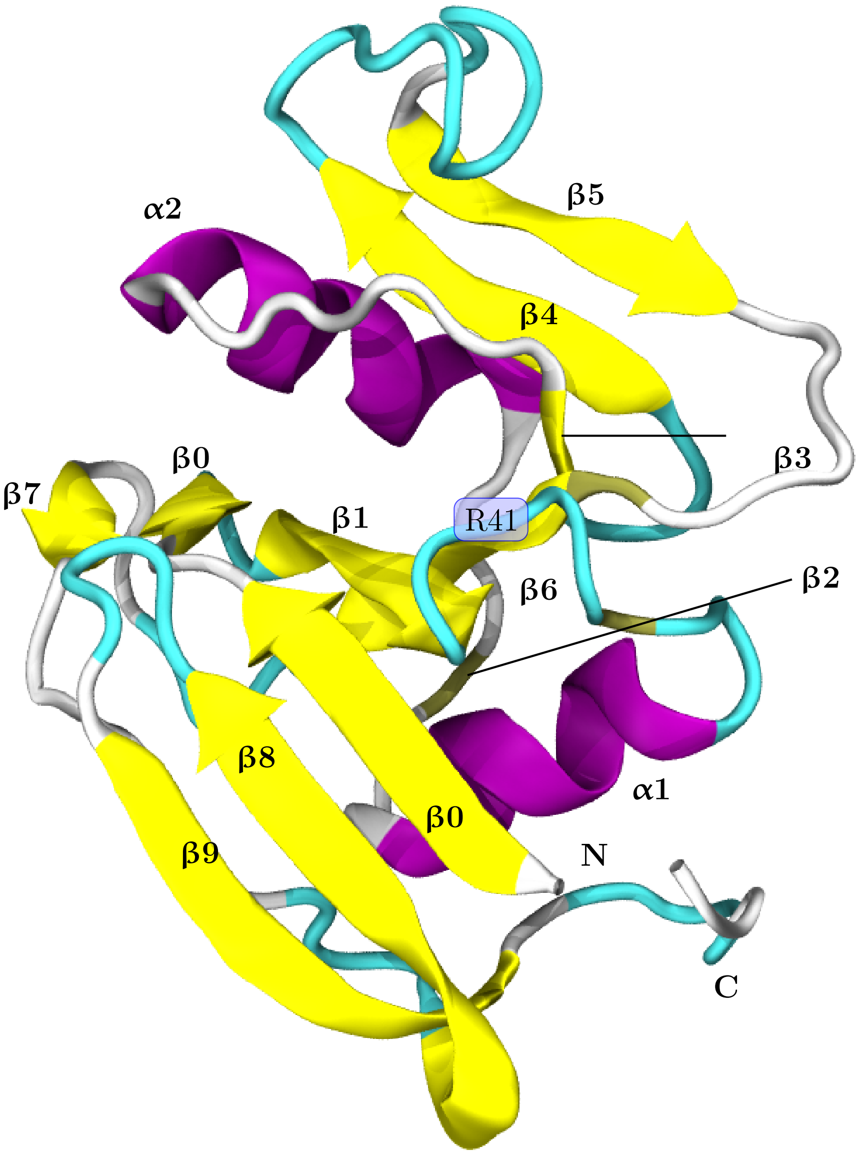


Figure A: Tertiary structure of the CD44 HABD domain. The positions/domains indicated in the image include the C- and N-termini, the secondary structure elements, and R41.

# 2 Methods

## 2.1 Descriptions of systems and simulations

**Table A: List of simulated systems. The first column gives the abbreviation for each simulation set. The second column describes the molecular content of each system. The third column gives the binding mode(s) of the CD44–HA complex that are found in a given system. The fourth column lists the duration of the simulations; some of them were repeated several times as indicated in the table.**

| Set | Contents | Binding modes | Duration (ns) |
|---|---|---|---|
| Seeding | CD44, $HA_{16}$ | Parallel | 1000 |
| Seeding | CD44, $HA_{16}$ | Crystallographic, upright | 2800 |
| Seeding | CD44, $HA_{16}$ | Parallel | 1000 |
| Seeding | CD44, $HA_{16}$ | Parallel | 1000 |
| Unbound | CD44, $HA_{18}$ | Parallel, upright | $5 \times 2000$ |
| Gathering | CD44, $HA_{16}$ | Crystallographic (B-form) | $3 \times 1000$ |
| Gathering | CD44, $HA_{16}$ | Parallel | $3 \times 1000$ |
| Gathering | CD44, $HA_{16}$ | Upright | $3 \times 1000$ |
| Gathering | CD44, $HA_{16}$ | Crystallographic (A-form) | 300 |
| Free energy | CD44, $HA_8$ | Crystallographic (B-form) | $32 \times 100$ |
| Free energy | CD44, $HA_8$ | Parallel | $35 \times 100$ |
| E-field weak | CD44, $HA_{16}$ | Crystallographic (B-form) | $20 \times 200$ |
| E-field weak | CD44, $HA_{16}$ | Parallel | $20 \times 200$ |
| E-field weak | CD44, $HA_{16}$ | Upright | $20 \times 200$ |
| E-field strong | CD44, $HA_{16}$ | Crystallographic (B-form) | $20 \times 20$ |
| E-field strong | CD44, $HA_{16}$ | Crystallographic (A-form) | $20 \times 20$ |
| E-field strong | CD44, $HA_{16}$ | Parallel | $20 \times 20$ |
| E-field strong | CD44, $HA_{16}$ | Upright | $20 \times 20$ |
| Clustering | 2xCD44, $HA_{64}$ | Parallel, crystallographic | $2 \times 3000$ |

Descriptions of all simulated systems can be found in Table A. The so-called 'Seeding' simulations entail four microsecond-scale simulations, as depicted in Table A. They contain one CD44-HABD and $HA_{16}$ oligomer, embedded inside a water box with a physiological (150 mM) concentration of NaCl. In these simulations, the HA ligand is placed close to the binding groove, engaging into either the parallel or the crystallographic binding mode soon after the simulation started. The main idea of this simulation set is to assess whether HA

will bind HABD spontaneously with the crystallographic mode described in the literature. We also ran a complementary simulation set (five replicas) called 'Unbound', where the protein and the ligand were 4 nm apart from each other in the beginning of the simulations (i.e., they were free to move relative to each other). The purpose of these simulations was to confirm that the binding site is readily accessible for the HA ligands even without any external guidance partly provided in the 'Seeding' simulation set.

In the 'Gathering' simulations, we studied three model systems, one for each binding mode. Each of these modes then had three replicas with different initial velocities. In all the cases, the same binding mode was sampled from the beginning of the equilibrium simulation to the end, thus yielding microseconds of quantitative statistics for each binding complex. The starting frames were extracted from the appropriate 'Seeding' simulation set. These initial frames were chosen because they represent a form of stable binding, i.e., the observed binding complexes lasted at least 100 ns in the 'Seeding' simulations. The first model system included the CD44-HABD–$HA_{16}$ complex in the crystallographic binding mode, with the side-chain of R41 in the "closed" conformation (type B-form[S2]), which enables it to form hydrogen bonds with the bound HA. The second and third systems displayed the parallel and the upright binding modes, respectively. In both cases, the R41 side-chain lied in the "open" conformation (type A-form[S2]). We also considered a fourth simulation system of the crystallographic binding complex in the A-form to serve for comparison. All three binding modes are thoroughly described in the Results section in the main text and in Section 3.

The so-called 'E-field weak' and 'E-field strong' simulation sets are abbreviations for *in silico* electrophoresis. The idea here was to measure the relative binding affinities of different CD44-HABD–HA complexes by using electric field to separate the two species, analogously to previous electrophoresis experiments used to determine the HA–CD44 binding affinities[S3]. The operation principle is simple; by restraining the protein, the negatively charged HA ligand is the only component able to move freely along the applied electric field. Both *in silico* electrophoresis sets were meant to assess the relative strength of each binding mode.

We used the same starting frames for these as for the previous 'Gathering' simulations. In the 'E-field strong' simulations, besides applying a stronger electric field, we analyzed one extra system: the crystallographic complex in the A-form (i.e., in the 'open' conformation). Overall, each binding mode was simulated 20 times, with each individual simulation spanning either 500 ns in the 'E-field weak' set or 20 ns in the 'E-field strong' set (Table A).

In the 'Free energy' simulations, we employed the umbrella sampling technique to measure the binding free energies of the crystallographic and parallel binding modes. In both modes, the HA lied in the same binding groove, so free energy differences between the two modes can reveal details of the specificity of the binding. Here we used the same respective starting frames as in the previous simulation set ('E-field weak') as initial structures for pulling the ligand away from the protein–ligand complex.

The 'Clustering' simulations included two systems with two CD44-HABDs and one long $HA_{64}$ polymer in each system. In the glycocalyx of a cell, HA is likely to interact in several CD44-HABD complexes simultaneously[S4]. This alone may change the preferred binding mode. Furthermore, high molecular weight HA has been reported to induce CD44 aggregation[S5,S6]. Our underlying idea with these two systems was, therefore, to simulate CD44-HA interaction in a more realistic, crowded environment and evaluate these two hypotheses. These systems allowed us to consider if the CD44-HABDs are able to diffuse along the rod-like HA strand and also to observe what binding modes spontaneously arise in such a case.

## 2.2 System construction

To construct the systems, we downloaded the crystal structure of human CD44-HABD Teriete et al.[S1] from the Protein Data Bank (RCSB) with the identifier 1UUH. After this, the construction involved the following steps. First, we removed the second monomer and all the water molecules from the model in order to have only one CD44-HABD protein. Second, we replaced the two selenomethionine residues at positions 63 and 66 with normal methionine

residues, because the former were incorporated into the protein structure solely for X-ray crystallography. We then used the GROMACS tool PDB2GMX to obtain a topology for the CD44-HABD for the AMBER99SB-ILDN[S7] force field. All HA structures and topologies for the corresponding GLYCAM06h[S8] force field were, on the other hand, built with a custom-made python script. This script attaches monosaccharides sequentially in order to generate more complex polymers, such as HA, simultaneously generating proper GROMACS topologies for them. All the generated HA structures were visually inspected to confirm the correct structure and the stereochemistry of the carbohydrate rings and linkages. All the system structures and corresponding topologies are publicly available in 'Zenodo.org'.

After generating the CD44-HABD and HA, we placed the CD44-HABD to the center of a simulation box of $10\,\mathrm{nm} \times 10\,\mathrm{nm} \times 10\,\mathrm{nm}$ (single protein systems). This box size was big enough to accommodate the globular CD44-HABD ($\approx$4 nm diameter) and the roughly 8.5 nm long $HA_{16}$ oligomer. In the 'Gathering', 'Electric field', and 'Free energy' sets HA was placed as already complexed with CD44-HABD in a particular binding mode. In the 'Seeding' set, HA was placed in contact with CD44-HABD, close to the binding groove but *a priori* not displaying any of the reported binding modes. Finally, in the 'Unbound' setup, HA was initially placed 4 nm from the CD44 active surface so it could freely diffuse before attaching. This set-up minimized the bias in the obtained binding modes. We then added explicit water molecules (TIP3P[S9]), neutralized the system, and added 150 mmol/l of sodium chloride ($Na^+Cl^-$[S10]), which reflects the conditions of the extracellular environment, such as blood plasma.

## 2.3 Parameters for systems with two CD44 HABDs and one HA polymer

In the 'Clustering' systems, we placed two CD44-HABDs to a rectangular box ($30\,\mathrm{nm} \times 8\,\mathrm{nm} \times 8\,\mathrm{nm}$) together with a $HA_{64}$ polymer ($\approx$30 nm long), spanning across the most of the $x$-direction of the simulation box. The two proteins were separated by 15 nm along the $x$-direction of

the box, thereby achieving maximum separation between them. Simulating systems such as this, with a size of 197,544 atoms for 3 μs per replica, requires substantial amounts of computational resources, meaning that larger setups without the rectangular box would not be feasible. To allow the usage of the rectangular box, both CD44-HABDs and HA had their movement partially restricted by position restraints.

In the case of the $HA_{64}$ polymer, both of its tail-ends were restrained in all three dimensions. More precisely, the polymer was restrained from the O1 oxygen of GlcNAc in the reducing end and from the O4 oxygen of GlcUA in the non-reducing end. These atoms were chosen as they are the farthest heavy atoms in an elongated HA strand. Both of these restraints are $10\,\mathrm{kJ\,mol^{-1}\,nm^{-2}}$ in the $x$-direction (parallel to the HA strand) and $200\,\mathrm{kJ\,mol^{-1}\,nm^{-2}}$ in the $y$- and $z$-directions. They enforce the HA strand to mimic a rod-like polymer with reduced mobility. Importantly, the fixed distance between the ends was smaller (27.6 nm) than the one of a fully stretched HA of the same size (roughly 32 nm). This allows $HA_{64}$ to wobble substantially during the simulations and thereby to adapt a relaxed conformation upon binding to CD44-HABD.

The two CD44-HABDs in the system also have restraints. Namely, we allowed them to move only along the direction of the HA polymer (the longest dimension of the box). We also partially restricted their rotational movement. For this, we imposed two different constrains per each CD44-HABD. Namely, we fixed the $C_\alpha$ atoms of the HABD residues L52 and D140 on the $y$- and $z$-dimensions with $20\,\mathrm{kJ\,mol^{-1}\,nm^{-2}}$ and $200\,\mathrm{kJ\,mol^{-1}\,nm^{-2}}$ position restraints, respectively. The milder (L52) restraint kept the protein from tilting too much, while the latter fixed its position firmly from a "bottom" residue (D140), emulating its attachment to a biological membrane via the stalk and transmembrane domains.

## 2.4 Special parameters for biased simulations

For the potential of mean force ('Free energy') and the *in silico* electrophoresis ('E-field') experiments, designed to estimate the binding free energy of HA to CD44-HABD, we needed to

fix the CD44-HABD position without significantly disturbing the HA binding site. Hence, we applied position restraints to the $C_\alpha$ atoms of residues T47, F56, and Q65 ($1000\,\mathrm{kJ\,mol^{-1}\,nm^{-2}}$). These residues were chosen as they are in a rather stiff region, form a large equilateral triangle, are buried inside the protein, and are located far enough from any crucial binding residues. We inspected all simulations visually to ensure that no anomalies were created due to these restrains. Finally, the protein binding site in the 'Free energy' simulations was oriented to the positive $x$-direction used to pull HA, while in the 'E-field' simulations we positioned the protein with the ligand-occupied binding site facing the negative $x$-direction (i.e., the "positive electrode" in our set-up). These orientations ensured that the HA ligand was able to detach into the water phase when the force (i.e., the umbrella potential or electric field) was applied.

In the umbrella sampling simulations, the HA ligand was pulled away from the binding groove to obtain a biased detaching event. This can be used to measure the strength of the binding in a given HABD–HA complex. First, we generated configurations of the $HA_8$ detachment process in both crystallographic and parallel modes by applying a harmonic biasing potential to the center of mass of the ring atoms (C1, C2, C3, C4, C5, and O5) of a stably bound GlcNAc (0) which forces the $HA_8$ to increase its distance to the binding groove, $\xi$. More specifically, we employed 40 ns simulations to pull the $HA_8$ ligand along the reaction coordinate ($\xi$) with a force constant of $1000\,\mathrm{kJ\,mol^{-1}\,nm^{-2}}$ and a pulling rate of $0.1\,\mathrm{nm\,ns^{-1}}$. The reference pull group in the CD44-HABD was selected to be the backbone of residues E75 to Y79. These amino acids are buried inside the "back wall" of the binding groove and their fluctuations are, therefore, minimal compared to other residues at the surface of the same site. From this pulling simulation, we extracted starting structures for free energy calculation using umbrella sampling spaced $0.10 \pm 0.01\,\mathrm{nm}$ apart along the $x$-axis. This distance was chosen to achieve proper sampling along the whole reaction coordinate $\xi$ when using a force constant of $1000\,\mathrm{kJ\,mol^{-1}\,nm^{-2}}$. In each window, the HA oligomer was constrained only in the $x$-direction, and was thus allowed to move freely in the corresponding $yz$-plane. The

pull groups were exactly the same as in the previous pulling simulation. From the 100 ns sampling per window, only the last 80 ns were used for the actual free energy calculation, and the leading 20 ns were regarded as equilibration. These time scales are essentially consistent with the current recommendations for equilibration and analysis times in umbrella sampling simulations, and while deviations from fully converged data are possible, they are expected to be minor given that the simulation results are in line with experiments (see discussion in the main paper).

In the strong and weak electric field simulations, we adjusted the field strengths to $17.6\,\mathrm{V\,nm^{-1}}$ and $1.76\,\mathrm{V\,nm^{-1}}$ in the $x$-direction and set the simulation timescale to 20 ns and 500 ns, respectively. Otherwise, the simulation parameters were identical to the ones in the unrestrained simulations described above. With these electric field strengths, we were able to see detachments of HA even from the crystallographic complex, which displayed the strongest HABD–HA interaction. On the other hand, the field strength was weak enough for some HA ligands to stay bound in the weaker HABD–HA complexes, allowing us to effectively compare the different cases. This can be seen by monitoring the minimum distance between the $C_\alpha$ atom of R78 and the methyl carbon of bound GlcNAc residue (GlcNAc(0)) in Figure B. These atoms were chosen, because they locate within 2 nm of each other in all the binding complexes. Furthermore, R78 resides in the "back wall" of the binding groove and its fluctuations are relatively low.

The field strengths used were above the breakdown field strength of pure water ($\sim$0.07 V/nm). Therefore, the simulations' conditions are not accessible experimentally and affect the water structure around HA and CD44. In particular, the application of these electric fields causes changes in the orientation of water molecules. These changes are calculated in Figure C. While the strong field clearly forces the water dipoles to orient along the field, the weaker field displays a significantly less pronounced effect. However, the qualitative observations measured with both of these fields are identical (see main text), thereby suggesting that water orientation does not significantly alter the relative affinities between the different binding

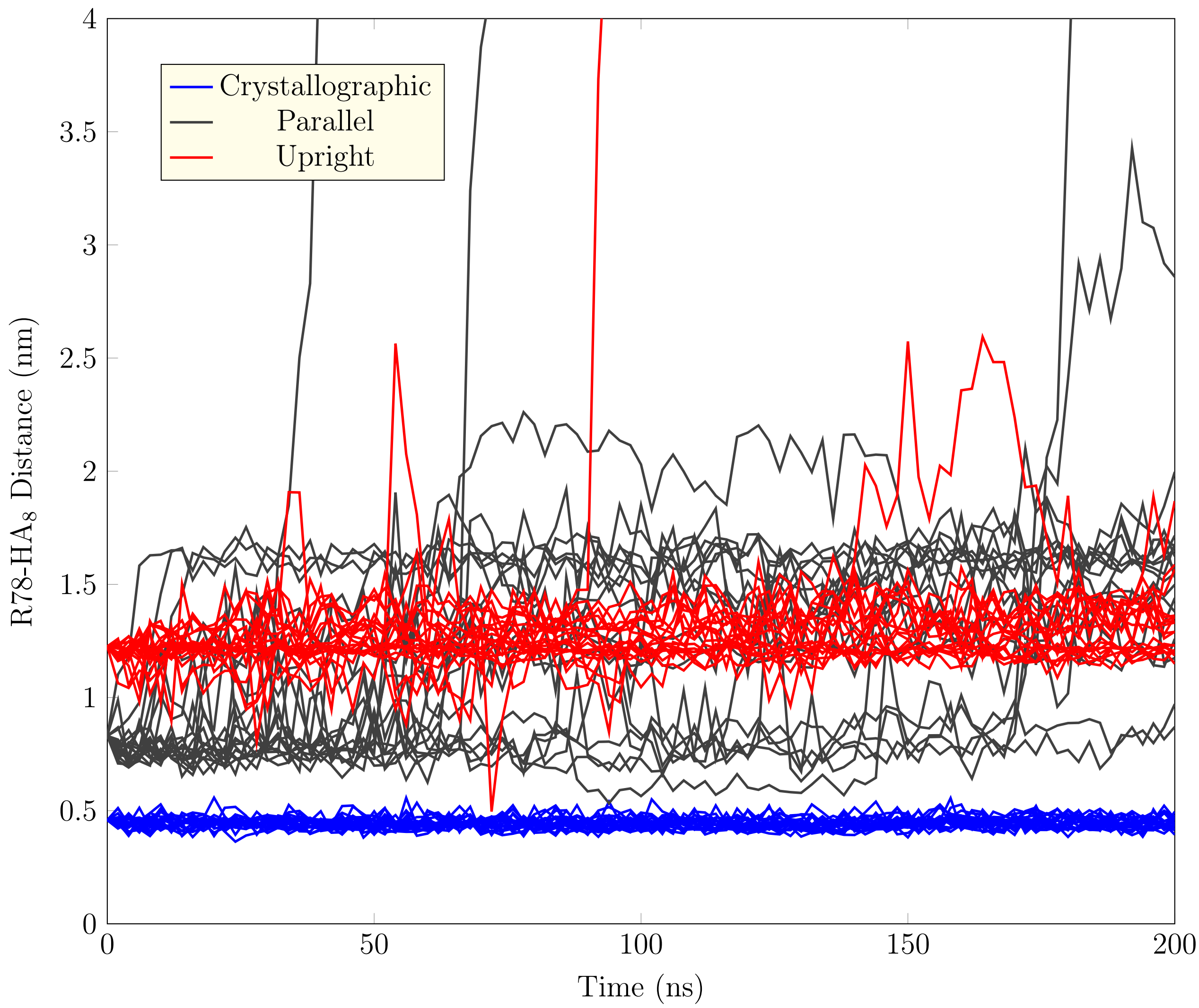


Figure B: Distance between the $C_\alpha$ atom of R78 respect the methyl carbon of the bound GlcNAc(0) residue as a function of time. Data are calculated from 20 simulation trajectories for each binding mode ('E-field weak' in Table A).

modes. This renders the results of these simulations qualitative. Using smaller fields would have required significantly longer simulations, which are not feasible.

## 2.5 Analysis details

Errors are reported with $t$ distribution (see Ref.[S11] for more details), where $e = ts/\sqrt{n}$ is the margin of error. Parameter $n$ is the number of samples, $s$ is the sample standard deviation, and $t$ is taken at a 95% confidence interval with $n - 1$ degrees of freedom. In a

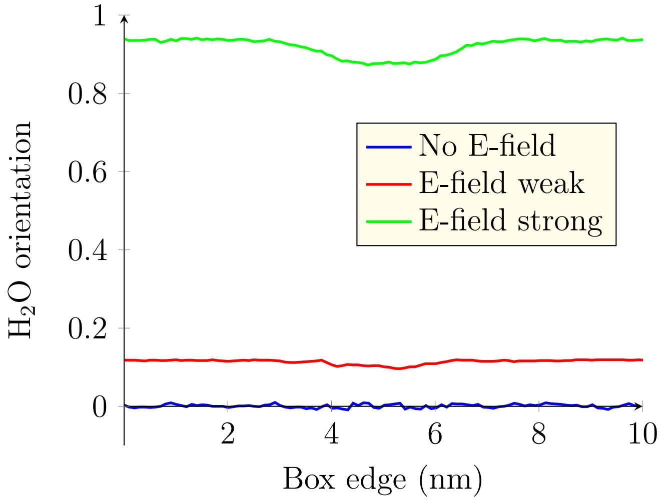


Figure C: Water orientation with respect to the normal of the box edge in the direction of the electric field. Data are calculated from the crystallographic 'Gathering' simulation set, the crystallographic 'E-field weak', and the crystallographic 'E-field strong' set. Other systems/replicas displayed similar behavior. The GROMACS tool `g_h2order` was used to calculate these data.

given system, each of the replica simulations is considered as an individual sample, so that for the binding simulations $n = 3$; $t = 4.303$, and for the *in silico* electrophoresis runs $n = 20$ and $t = 2.093$[S11].

# 3 Detailed description of Hyaluronan–CD44 binding modes

Figure D shows a zoomed view of the main binding residues of HA on the CD44 surface.

To characterize and compare the binding modes, several properties were extracted and averaged from each corresponding replica simulation. To begin with, we computed pairwise contacts between CD44 and HA, see Figure E. A contact is defined as two particles being within 0.6 nm or less from each other, so that a given atom can establish more than one contact simultaneously. This representation provides a list of CD44 residues potentially significant for each binding mode.

To further characterize the interactions observed in our 'Gathering' simulations, we separated the contributions of interactions by type, repeating the contact analysis (see the main

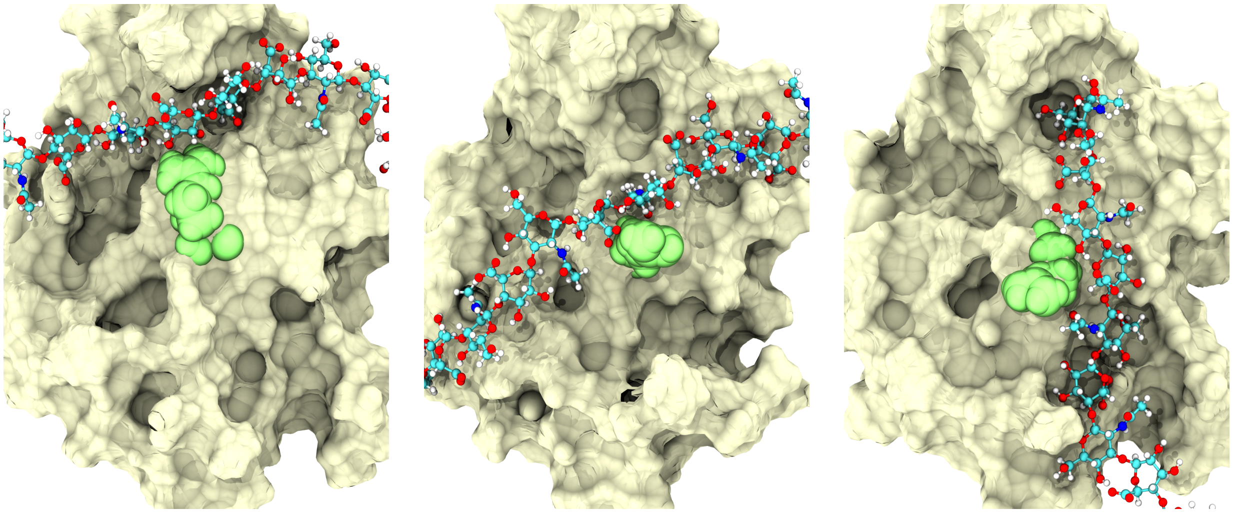

Figure D: Three binding modes in a close view. Tan surface represents the CD44 HABD domain, the light green spheres depict R41 (as the most important binding residue), and the multicolored ball-and-stick representation is the $HA_{16}$ oligomer.

text) with atoms grouped according to their chemical nature: charge, hydrophobic, hydrogen donor/acceptor, backbone, and all the leftovers. Detailed information of this analysis can be found in Tables B, C, and D.

Figures F, G, and H present the contact information in more detail, splitting also HA into residues (residue-by-residue maps).

We also computed hydrogen bonds between the HA polymer and the amino acid residues of CD44 (Figure I). This shows that hydrogen bonds are key interactions affecting strength and specificity of the HA–CD44 binding.

## 3.1 Crystallographic mode

As described in Banerji et al.[S2], the recognition of HA in the crystallographic binding complex is facilitated mostly by hydrogen bonds involving carboxyl and N-acetyl groups of HA (Figure I). Furthermore, the final recognition mostly rests on just three HA carbohydrate units attached to the pincer-like binding groove of CD44. They display extremely low mobility during their attachment in the crystallographic complex, indicating a specific and strong interaction, see Figure 4 in the main text. In our representative structure, these carbohydrate

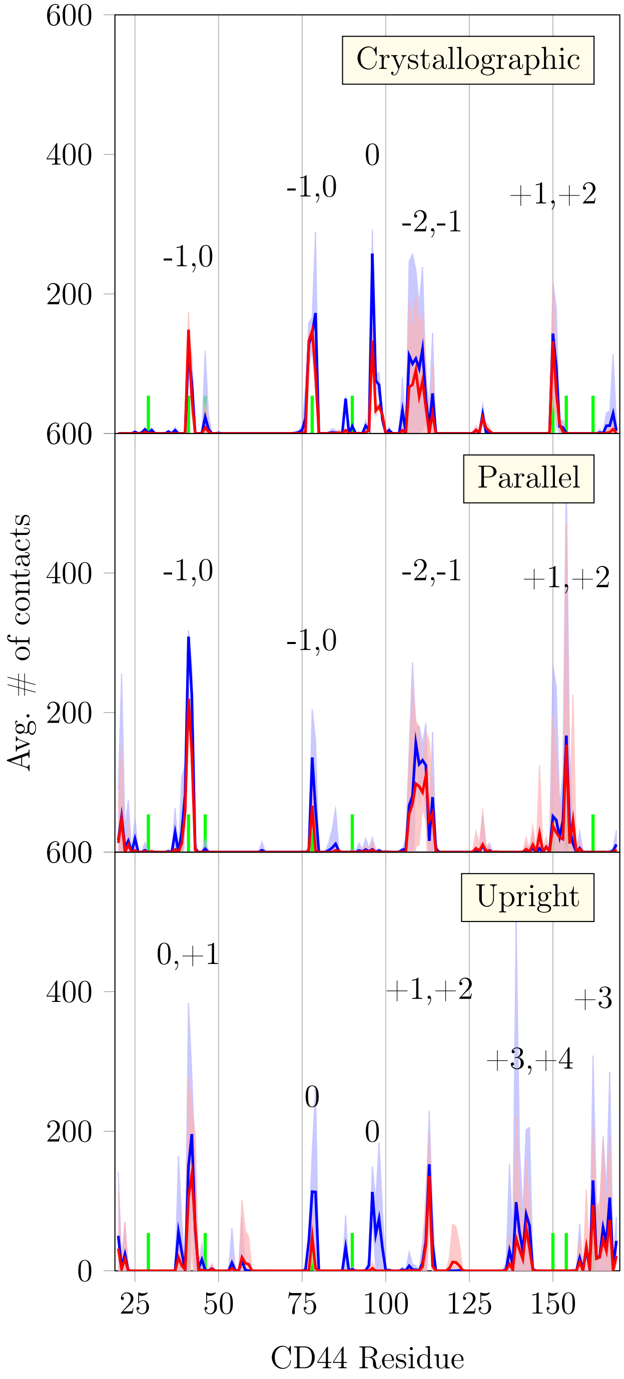


Figure E: Average number of contacts of HA GlcNAc and GlcUA residues with each residue in the CD44 HABD, calculated from three replica systems ($3 \times 1000\,\mathrm{ns}$) per each binding mode (Gathering simulations in Table A). Errors ($t$ distribution, 95 % confidence interval) are shaded accordingly. Blue color represents hydrogen bonds between CD44 and GlcNAc residues, while red color represents hydrogen bonds with GlcUA residues.

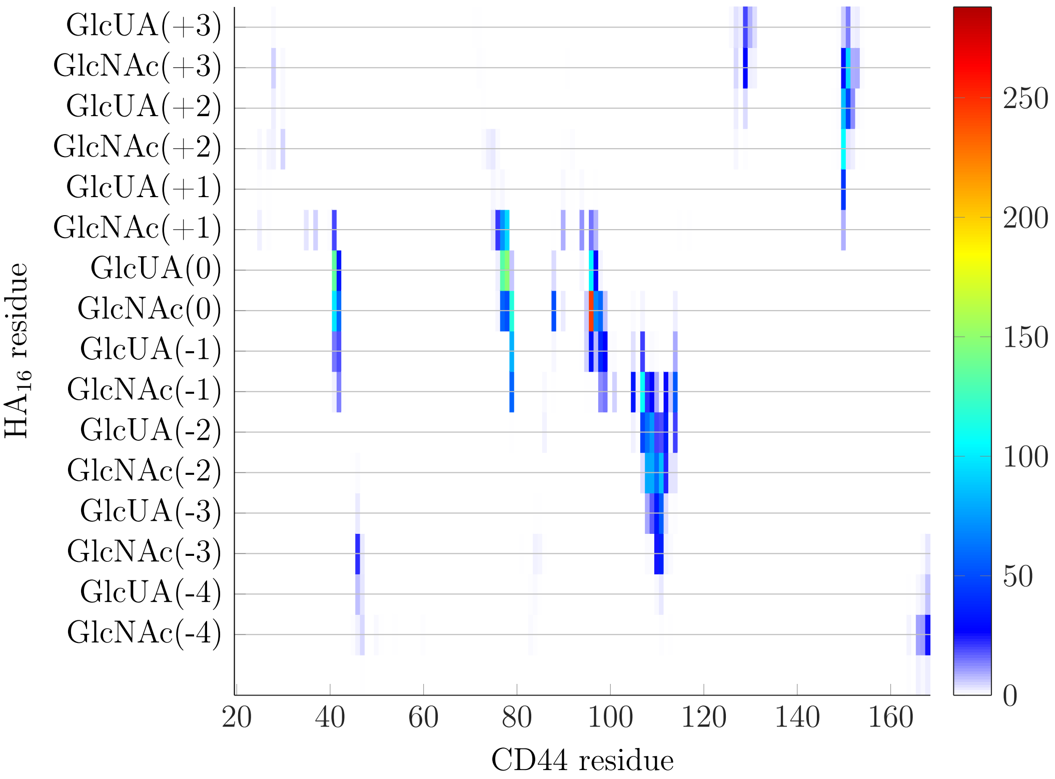


Figure F: Average number of contacts (pixels) in the crystallographic binding complex for each HA monomer (vertical axis) versus each CD44 HABD residue (horizontal axis). Data is calculated from the Gathering simulations, listed in Table A.

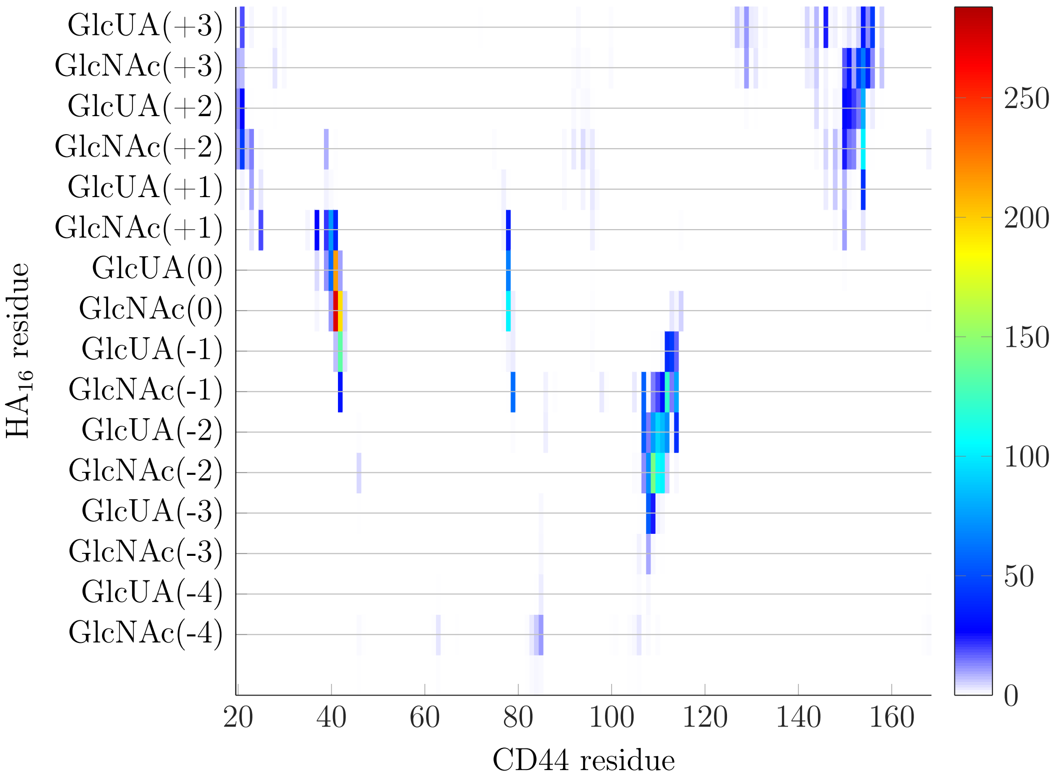


Figure G: Average number of contacts (pixels) in the parallel binding complex for each HA monomer (vertical axis) versus each CD44 HABD residue (horizontal axis). Data is calculated from the Gathering simulations, listed in Table A.

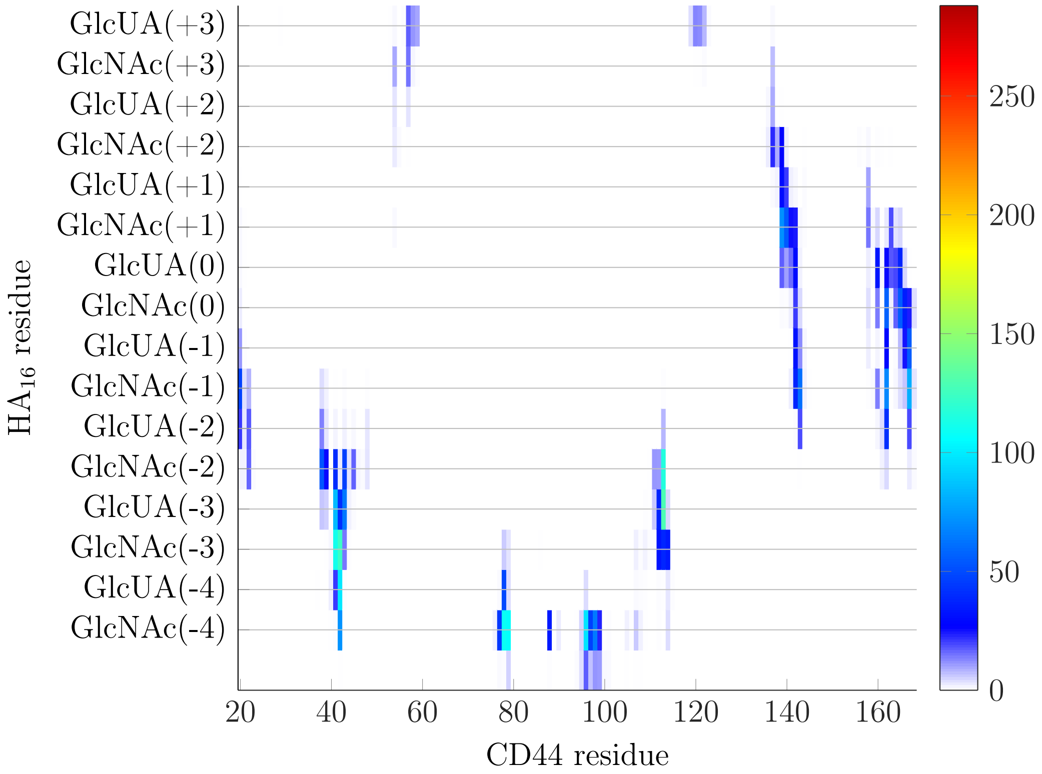


Figure H: Average number of contacts (pixels) in the upright binding complex for each HA monomer (vertical axis) versus each CD44 HABD residue (horizontal axis). Data are calculated from the Gathering simulations listed in Table A.

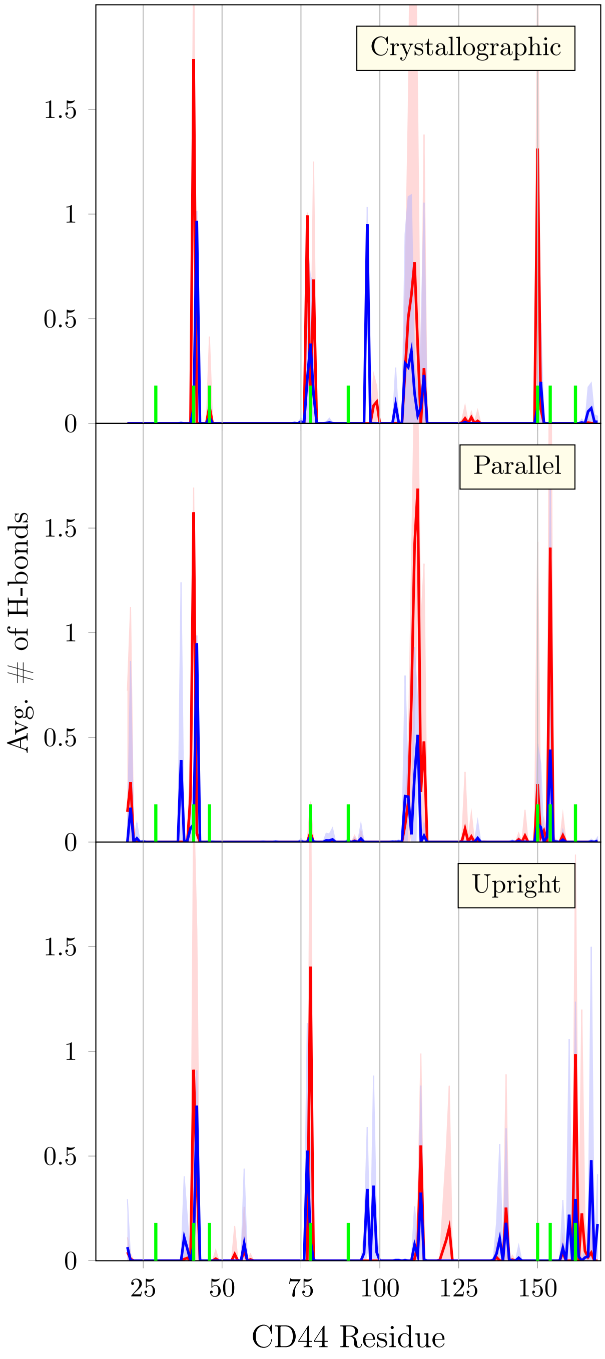


Figure I: Average number of hydrogen bonds of HA GlcNAc and GlcUA residues with each residue in the CD44 HABD, calculated from three replica systems ($3 \times 1000\,\mathrm{ns}$) per each binding mode (Gathering simulations in Table A). Errors ($t$ distribution, 95 % confidence interval) are shaded accordingly. Blue color represents hydrogen bonds between CD44 and GlcNAc residues, while red color represents hydrogen bonds with GlcUA residues.

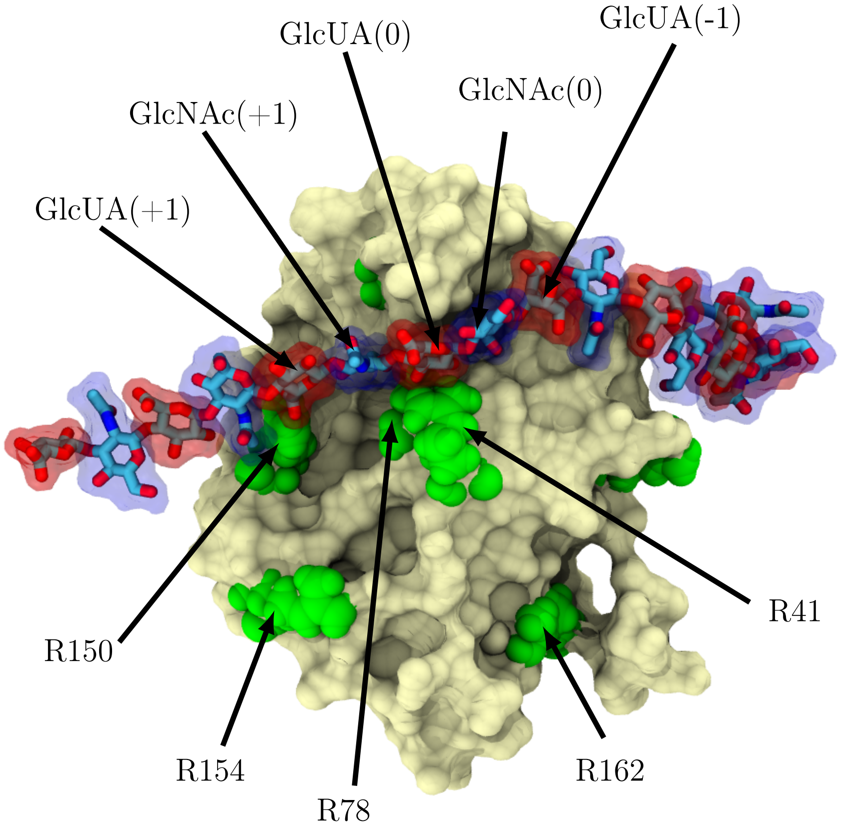


Figure J: Naming of HA residues in our simulations of the crystallographic binding complex. The tan surface represents the protein in the first frame of the trajectory, whereas the green spheres are the arginine residues. The $HA_{16}$ oligomer is depicted with a liqourice-like structure, where blue represents carbon and red oxygen. Furthermore, the GlcNAc residues are shaded with blue and GlcUA residues with red. For clarity, only a few of the HA saccharides and CD44 arginines are shown with the black arrows.

units are designated as GlcUA(0), GlcNAc(0), and GlcUA(-1) as shown in Figure J.

First, one of the carboxyl oxygens of GlcUA(-1) forms a strong hydrogen bond with the hydroxyl group of Y79. The carboxyl group of GlcUA(-1) also forms hydrogen bonds with the backbone amino group of A98 and A99. Second, the carbonyl oxygen of the amide in GlcNAc(0) forms hydrogen bonds with the hydroxyl group of Y42 and the guanidinium group of the side-chain of R41. The GlcNac(0) residue is further recognized through its methyl group, which lies in a hydrophobic pocket lined mainly by the side-chains of C88 and Y79. The sugar ring of GlcNAc(0) also interacts hydrophobically with the side-chain of I96. Lastly, GlcUA(0) forms two frequent hydrogen bonds with the guanidinium group of R41 through its O2 and O3 hydroxyl oxygens. These oxygens bind also to the underlying R78, so that they are constantly bound to either R41 or R78.

In addition to the aforementioned interactions with the residues that form the pincer-like area in the main binding groove, HA residues GlcUA(-3) to GlcNAc(-1) form multiple hydrogen bonds with a stretch of amino acids ranging from the residue 108 to 114 (T108, S109, N110, T111, S112, Y114). These interactions are more short-lived and they experience more variance between, and within, the replica systems compared to the interactions in the binding groove. However, they seem to provide additional stabilization to the crystallographic CD44–HA complex.

Apart from the methyl group of GlcNAc(0), the most important hydrophobic contacts are the interactions between the sugar ring of GlcNAc(-1) with hydrophobic side-chain of L107 and the sugar ring of GlcUA(0) with both C77 and I96.

Notably, the crystallographic binding complex lacks charged interactions almost entirely. In fact, the carboxyl group of GlcUA(+1) is the only HA residue forming a salt bridge, albeit dynamic, with R150. Detailed description of the contacts can be found in Table B.

We also analyzed the orientation of HA in the crystallographic mode, see Figure K. Clearly, the alignments of disaccharide(0) and GlcNAc(+1) are the key structural features of this binding mode. All replicas consistently show the same orientation for these monomers

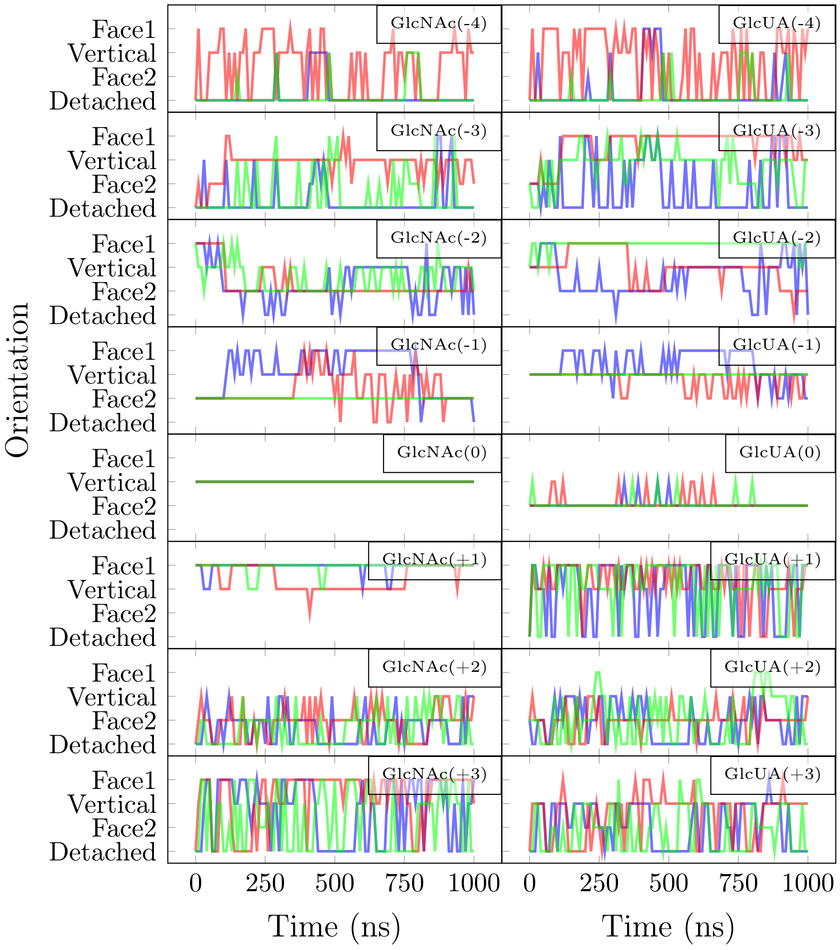


Figure K: Orientation of the carbohydrate monomers of HA as a function of simulation time. 'Face1' corresponds to the face of the monosaccharide, where the H2 and H4 hydrogens point to. 'Face2' corresponds to the opposing face of the monosaccharide, where the H1, H3, and H5 hydrogens point to. 'Vertical' orientation is a conformation between the two faces. 'Detached' means that the monosaccharide is further than 1 nm from the protein surface. The names of the monomers are given in the legends. These data are calculated from three replica systems ($3 \times 1000$ ns) of crystallographic binding mode simulations (Gathering simulations in Table A). The three colors – blue, green, and red, – represent the data for each replica. Data points were collected for every 10 ns in the trajectory.

with little deviation or detachments. In particular, GlcNAc(0) displays a unique orientation, as it sits firmly anchored to the protein with its N-acetyl group protruding to the hydrophobic pocket lined by the side-chains of C88 and Y79. While we observe more detachments further along the HA strand, particularly between GlcUA(+1) and GlcUA(+3), the carbohydrate ring orientation during their interaction remains highly specific. This signals the presence of specific interactions between HA and CD44, regardless of their transient life time (Figure 4 in the main text). This behavior is shared by the other end of the oligomer as well, although both the attachment and detachment periods last longer. Notably, the section between GlcNAc(-1) and GlcAU(-2) requires special attention. This stretch presents mixed orientations of the carbohydrate rings when interacting with CD44, yet there are no detachments. Behavior like this denotes the existence of the important polar region next to the binding groove, which does not host any precise interactions, yet still has a considerable contribution to the energetics of this binding mode.

### 3.2 Parallel mode

One striking result of our simulations is the appearance of a binding mode not described previously in the literature. Interestingly, most of our unbiased simulations end up in this mode and then remain unchanged, suggesting it is rather stable in our simulation time scales. This parallel binding mode – despite resembling the crystallographic mode – is fundamentally different.

To begin with, the HA resides in the "lower" part of the binding groove with the carbohydrate rings left of R41 lying parallel to the surface of the protein, see Figures L and M. This is a radical change compared to the crystallographic mode, where the carbohydrates present a perpendicular alignment, see Figure J. Furthermore, while in the crystallographic mode GlcUA(0) is aligned on top of R41, in the parallel mode R41 is found between GlcUA(0) and GlcNAc(0). This accounts for an effective displacement of the whole HA by half a disaccharide, resulting in HA being slightly pushed out from the binding groove. This, in turn, leads

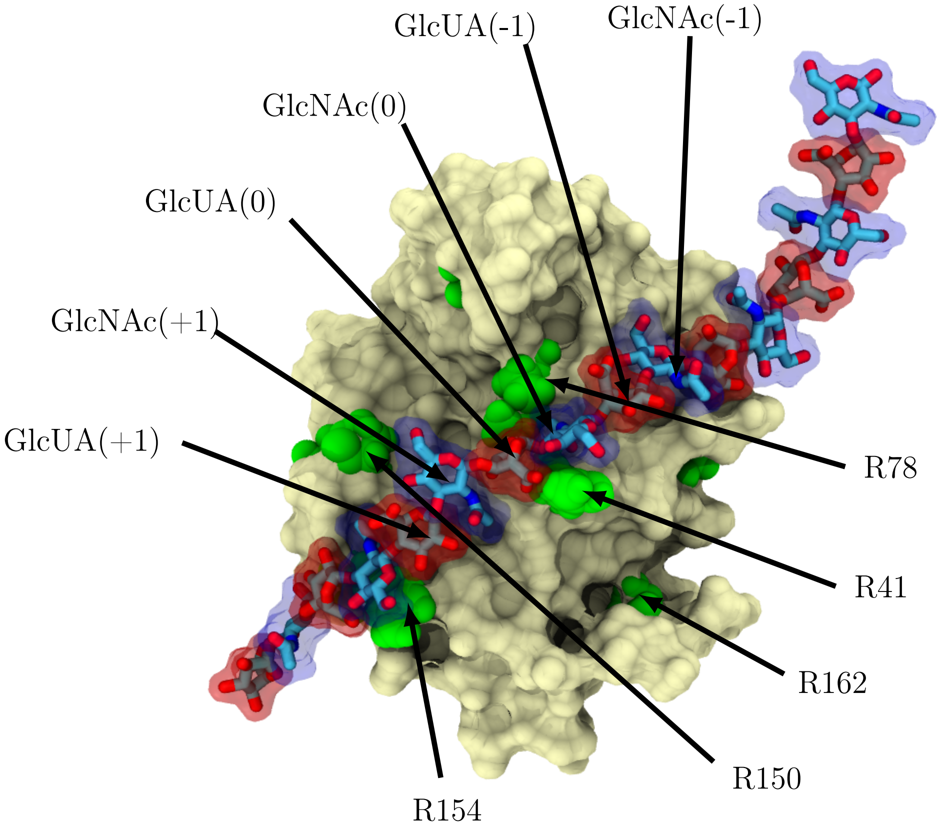


Figure L: Naming of HA residues in our simulations of the parallel binding complex. The tan surface represents the protein in the first frame of the trajectory, whereas the green spheres are the arginine residues. The $HA_{16}$ oligomer is depicted with a liqourice-like structure, where blue represents carbon and red oxygen. Furthermore, the GlcNAc residues are shaded with blue and GlcUA residues with red. For clarity, only a few of the HA saccharides and CD44 arginines are shown with the black arrows.

to a significantly decreased interaction with R78 and the surrounding amino acids that are highly relevant in the crystallographic mode. In the parallel mode, there are no hydrogen bonds within this region, suggesting that the binding would be weaker compared to the crystallographic mode. However, the carboxyl group of GlcUA(0) forms a strong salt bridge with the guanidinium group of the R41 side-chain, which is further stabilized by strong hydrogen bonds with R41 and Y42, similarly to the crystallographic mode. We should point out that the "B" form described in Ref. S2 and required by the crystallographic mode to establish hydrogen bonds with R41 is not possible in the parallel mode, as HA already sits on top of R41.

Another important contrast to the crystallographic mode is found in the end-part of the interacting segment of HA. The interaction with R150, found in the crystallographic mode, is mostly replaced by interaction with R154. This is achieved by tilting down the HA segment, so that it settles between R150 and R154. For this reason, the parallel mode can establish hydrogen bonds simultaneously with the guanidinium group of R154, and to a smaller extent with the guanidinium group of R150. Actually, a similar configuration was predicted by Teriete et al. employing the CD44 HABD crystal structure, where they predicted a binding complex that is a hybrid between the crystallographic form and our parallel form. It is important to mention that none of our simulations displayed such a hybrid binding mode. The positioning between the R150 and R154 also explains the lower mobility of HA between the binding groove and the R150–R154 region when compared to the crystallographic mode.

Finally, in the parallel mode, HA disaccharides (-1) and (-2) show multiple variable hydrophobic contacts with the amino acids T108, S109, N110, T111, S112, and Y114, as in the crystallographic mode (data not shown). These contacts are additionally stabilized by several polar contacts. In particular, the dynamic hydrogen bonds formed between the functional groups of GlcNAc(-1), GlcUA(-2), GlcNAc(-2), and GlcUA(-3) and protein residues S109, N110, T111, S112, and Y114 seem to be a key factor in the stabilization of this region.

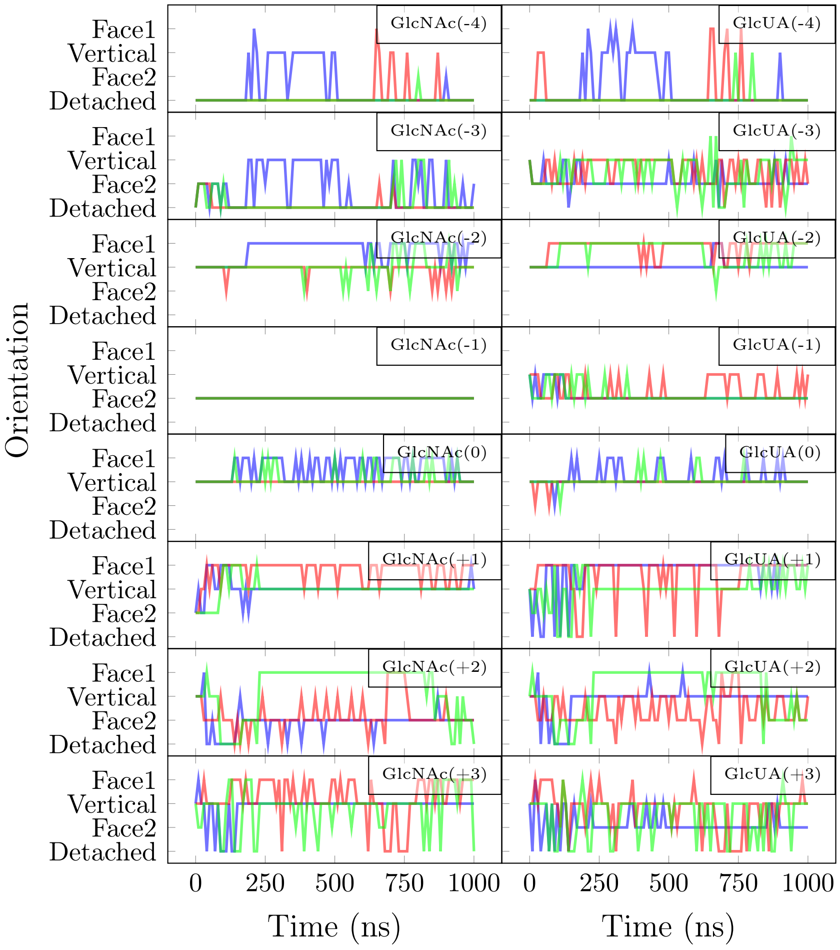


Figure M: Orientation of the carbohydrate monomers of HA as a function of simulation time. 'Face1' corresponds to the face of the monosaccharide, where the H1, H3, and H3 hydrogens point to. 'Face2' corresponds to the opposing face of the monosaccharide, where the H2 and H4 hydrogens point to. 'Vertical' orientation is a conformation between the two faces. 'Detached' means that the monosaccharide is further than 1 nm from the protein surface. The names of the monomers are given in the legends. These data are calculated from three replica systems ($3 \times 1000$ ns) of crystallographic binding mode simulations (Gathering simulations in Table A). The three colors – blue, green, and red, – represent the data for each replica. Data points were collected for every 10 ns in the trajectory.

Additional recognition is obtained from the hydrophobic interactions between the phenyl ring of Y42 and the sugar ring of GlcUA(-1). HA residues GlcUA(-2) and GlcNAc(-1) also form multiple labile hydrophobic contacts with the side-chain of L107. See Table C for a detailed description of the contacts in the parallel mode.

## 3.3 Upright mode

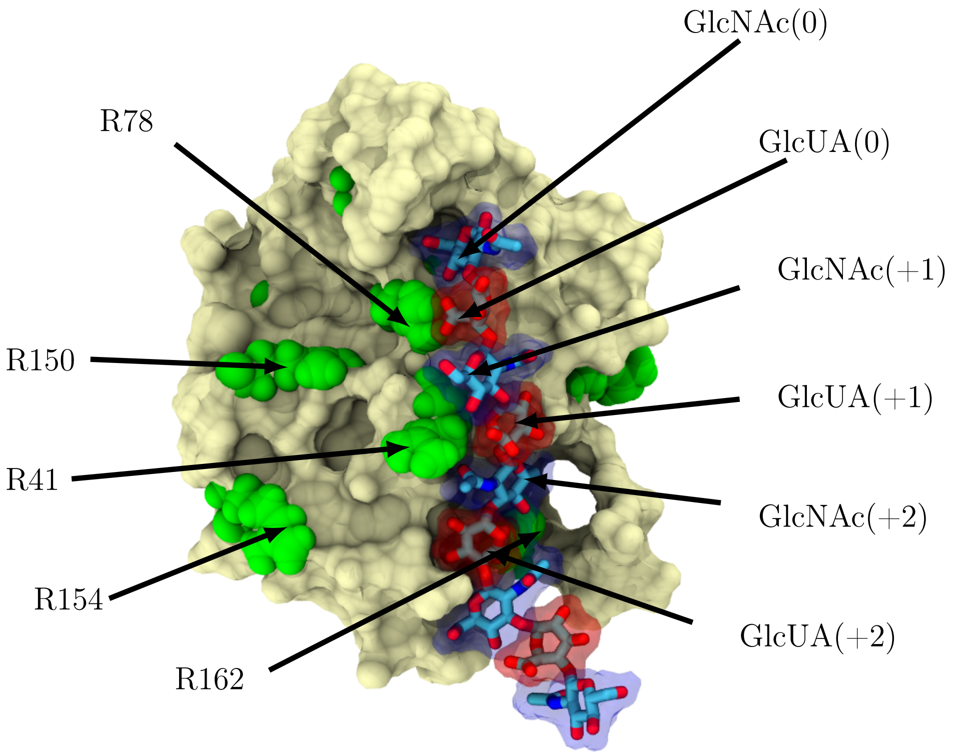


Figure N: Naming of HA residues in our simulations of the upright binding complex. The tan surface represents the protein in the first frame of the trajectory, whereas the green spheres are the arginine residues. The $HA_{16}$ oligomer is depicted with a liqourice-like structure, where blue represents carbon and red oxygen. Furthermore, the GlcNAc residues are shaded with blue and GlcUA residues with red. For clarity, only a few of the HA saccharides and CD44 arginines are shown with the black arrows. For the same reason, a total of seven last HA residues have also been cropped from this figure.

Our simulations also presented us with another binding mode in which the HA oligomer is, in essence, perpendicular to the other two modes, while sharing the interaction with R41, see Figure N. This mode was already suggested by Teriete et al. in Ref. S1 based on the

complementarity of HA to the CD44 crystal structure. However, neither experimental nor simulation confirmation of its existence was available.

Contrary to the previous binding modes, the upright mode does not have any carbohydrate unit which would interact with CD44 with its orientation fully fixed at all times, see Figure O. Still, roughly eight carbohydrate monomers, ranging from from GlcNAc(0) to GlcUA(+3), are stably interacting with CD44 and displaying preferential orientations on the protein surface, see Figure 4 in the main text. It is interesting though that some of the monomers, e.g., GlcNAc(+3), are found in stable but different orientations in separate replicas, indicating weak specificity for the binding.

HA lies in a mostly uncharged region of the binding surface, forming several short-lived and non-specific hydrophobic contacts with the HABD surface. Yet, some of the most prominent contacts are reasonably well conserved among the replicas. For instance, the sugar ring of GlcNAc(0) (HA residues named according to Figure N) forms extensive hydrophobic stacking with the phenyl ring of Y79. The next two HA residues, GlcUA(0) and GlcNAc(+1), form similar hydrophobic contacts with adjacent Y42 and, to a lesser extent, with the more buried Y114. The sugar rings of GlcUA(+1) and GlcNAc(+2), on the other hand, interact hydrophobically with Q113. Closer to the C-terminus of the protein, the HA residues GlcNac(+3) and GlcUA(+3) are occasionally stabilized by van der Waals interactions with D167.

Even with the lesser specificity, the alignment of the HA oligomer still seems to be heavily guided by salt bridges and hydrogen bonds, formed especially with R41, R78, and R162. Most notably, the C6 carbon of GlcNAc(0) faces the hydrophobic pocket lined by the side-chains of C88 and Y79, leaving the O6 hydroxyl oxygen to render one hydrogen bond with the backbone oxygen of C77 and one water-mediated hydrogen bond with the backbone oxygen of I96. Based on our simulations, it is unclear if this GlcNAc residue could be turned 180 degrees, placing the methyl group to the binding pocket and leaving the carbonyl oxygen to render hydrogen bonds with the backbone oxygens of C77 and I96.

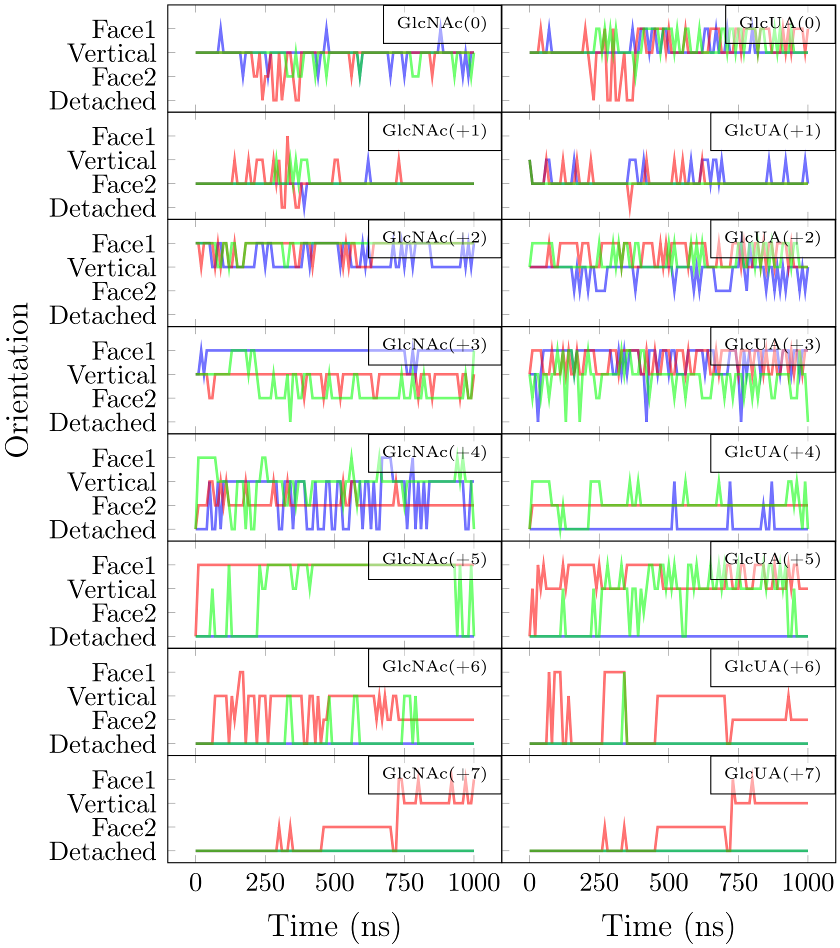


Figure O: Orientation of the carbohydrate monomers of HA as a function of simulation time. 'Face1' corresponds to the face of the monosaccharide, where the H2 and H4 hydrogens point to. 'Face2' corresponds to the opposing face of the monosaccharide, where the H1, H3, and H5 hydrogens point to. 'Vertical' orientation is a conformation between the two faces. 'Detached' means that the monosaccharide is further than 1 nm from the protein surface. The names of the monomers are given in the legends. These data are calculated from three replica systems ($3 \times 1000$ ns) of the crystallographic binding mode simulations (Gathering simulations in Table A). The three colors – blue, green, and red, – represent the data for each replica. Data points were collected for every 10 ns in the trajectory.

Similar orientation of the sugar ring is present in the crystallographic mode, hinting that it might be energetically more favorable. Furthermore, the carboxyl group of GlcUA(0) forms a salt bridge with the guanidinium group of R78, while GlcUA(+1) interacts electrostatically with the guanidinium of R41. The ring hydroxyls of GlcUA(+1) also form multiple short-lived hydrogen bonds with residues T111, S112, and Q113. Further along the HA strand, the carbonyl oxygen of GlcNAc(+2) renders one hydrogen bond to L38 nitrogen, while the carboxyl groups of GlcUA(+3) and GlcUA(+4) form hydrogen bonds and occasional salt bridges with the guanidinium group of R162. A detailed list of contacts in the upright mode can be found in Table D.

It should be noted that, in our structure, the HA oligomer was attached to the protein with its reducing tail-end. The complete picture of the binding complex might vary slightly if HA connects to CD44 with its middle part rather than just one of its tail-ends. However, all the spontaneously-formed upright complexes we observed were connected like described above.

# 4 A-to-B transitions in the crystallographic binding mode

Figure P shows the two possible conformations of R41 side-chain. Jamison *et al.* showed that this conformation change is induced by a shift in the $\phi$ backbone dihedral angle of Y42.[S12] Figure Q shows how this angle behaves in the second replica of the 'Seeding' simulations. In this system, A-to-B transition takes place while HA is bound in the crystallographic manner. The B-to-A transition, on the other hand occurs after the HA has been detached from the protein.

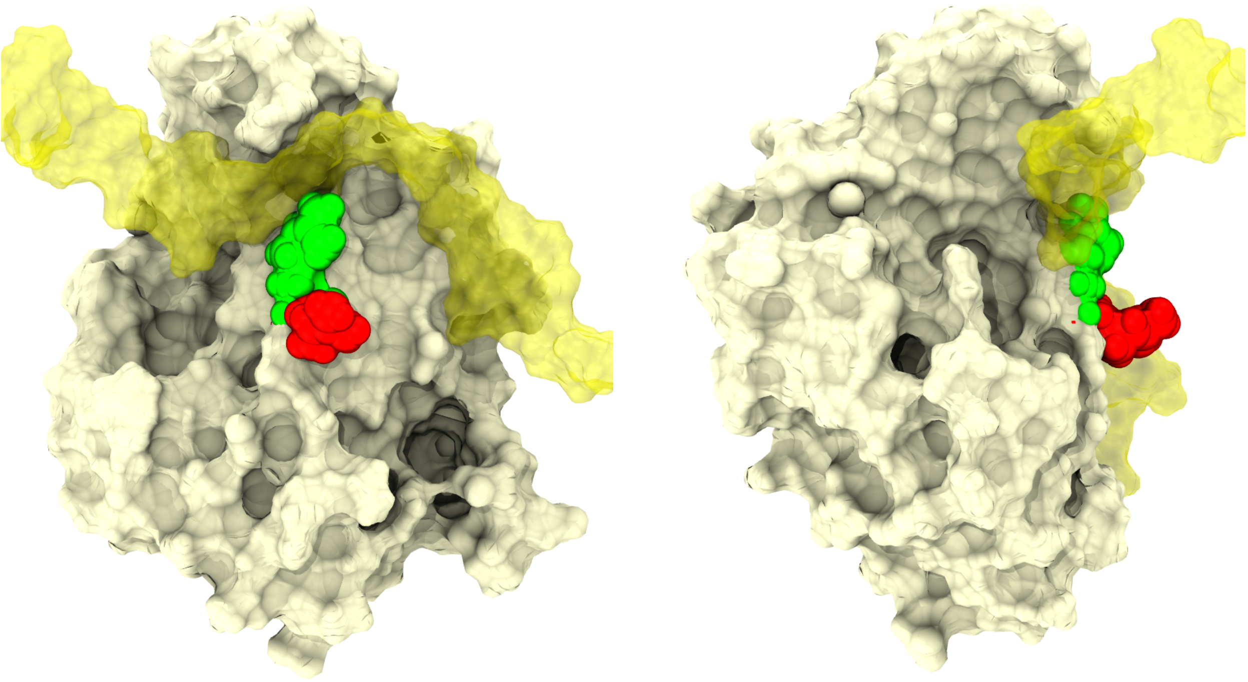

(a) Front view.

(b) Side view.

Figure P: Figures Pa and Pb depict the A-to-B conformational transition viewed from the 'front' and the 'side' of the protein molecule, respectively. The B-conformation (R41 side-chain is "closed") is shown in green, while the A-conformation (R41 side-chain is "open") is depicted red. CD44 is depicted as a pale surface and HA16 is shown as a yellow shadow.

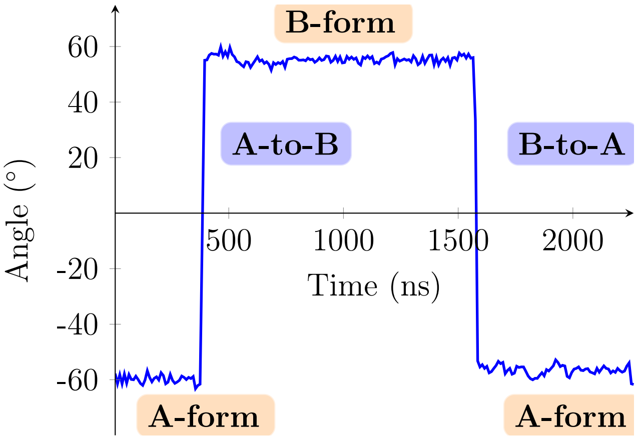


Figure Q: A-to-B-to-A transitions in the second replica of the "Seeding" simulations. Graph shows the $\phi$ backbone dihedral angle of Y42, which has previously been identified as the molecular switch between the A and B conformations.[S12] The first A-to-B transition takes place while HA is bound but the latter B-to-A transition happens when the HA is detached.

# 5 Appendix 1: table of contacts for each binding mode

**Table B: List of residues with the most contacts in the crystallographic binding mode. Calculated from the three replicas of the 'Gathering' simulations. 'Resid' column gives the number of the residue, 'All' stands for the number of all contacts, 'Hydrophob.' corresponds to contacts to hydrophobic parts of the protein, 'Charged' refers to contacts to the charged part of the proteins, 'CAHA' refers to contacts to the Cα and Hα atoms of the protein backbone, 'NH' is for contacts to the N and H atoms of the protein backbone, 'OC' provides data for the contacts to the O and C atoms of the protein backbone, and 'other' stands for contacts to all the remaining part of the protein residues. The values are numbers of contacts and the errors are standard deviations.**

| Resid | All | Hydrophob. | Charged | CAHA | NH | OC | Other |
|---|---|---|---|---|---|---|---|
| 41 | 286.36 ± 40.26 | 25.72 ± 11.70 | 216.04 ± 31.03 | 0.64 ± 1.54 | 0.92 ± 2.01 | 0.03 ± 0.21 | 43.01 ± 11.19 |
| 41 | 246.54 ± 38.08 | 19.71 ± 12.12 | 189.46 ± 19.02 | 0.38 ± 1.24 | 0.55 ± 1.62 | 0.01 ± 0.17 | 36.43 ± 13.20 |
| 41 | 266.88 ± 40.14 | 20.78 ± 10.89 | 208.35 ± 22.63 | 0.40 ± 1.17 | 0.54 ± 1.54 | 0.01 ± 0.09 | 36.79 ± 12.54 |
| 42 | 145.22 ± 69.20 | 90.36 ± 55.08 | 0.00 ± 0.00 | 1.85 ± 3.53 | 0.11 ± 0.39 | 1.68 ± 3.17 | 51.23 ± 8.35 |
| 42 | 98.02 ± 12.15 | 53.72 ± 10.13 | 0.00 ± 0.00 | 0.00 ± 0.00 | 0.00 ± 0.06 | 0.00 ± 0.00 | 44.29 ± 3.14 |
| 42 | 126.39 ± 20.95 | 76.09 ± 16.44 | 0.00 ± 0.00 | 0.00 ± 0.00 | 0.00 ± 0.00 | 0.00 ± 0.00 | 50.30 ± 5.39 |
| 46 | 3.11 ± 15.18 | 0.19 ± 1.71 | 2.71 ± 13.42 | 0.00 ± 0.04 | 0.04 ± 0.31 | 0.01 ± 0.10 | 0.16 ± 1.71 |
| 46 | 91.59 ± 104.63 | 17.48 ± 18.31 | 58.21 ± 87.21 | 0.55 ± 1.49 | 0.44 ± 1.65 | 0.87 ± 2.45 | 14.05 ± 15.98 |
| 46 | 2.07 ± 16.41 | 0.15 ± 0.91 | 1.88 ± 15.59 | 0.00 ± 0.00 | 0.00 ± 0.04 | 0.00 ± 0.04 | 0.04 ± 0.33 |
| 77 | 247.65 ± 39.87 | 94.65 ± 18.05 | 0.00 ± 0.00 | 36.85 ± 6.45 | 24.62 ± 10.22 | 54.63 ± 4.90 | 36.90 ± 4.83 |
| 77 | 272.85 ± 34.85 | 100.10 ± 12.67 | 0.00 ± 0.00 | 41.68 ± 6.71 | 33.37 ± 10.52 | 60.36 ± 7.13 | 37.34 ± 3.61 |
| 77 | 261.42 ± 38.14 | 99.61 ± 16.82 | 0.00 ± 0.00 | 38.78 ± 6.37 | 30.72 ± 10.48 | 55.55 ± 4.43 | 36.75 ± 4.81 |
| 78 | 283.82 ± 48.14 | 91.60 ± 22.03 | 66.38 ± 24.87 | 33.28 ± 2.65 | 23.82 ± 4.03 | 11.50 ± 1.83 | 57.23 ± 13.98 |
| 78 | 297.13 ± 42.36 | 104.58 ± 19.03 | 55.60 ± 22.12 | 35.31 ± 3.04 | 29.45 ± 6.46 | 11.64 ± 2.40 | 60.55 ± 11.70 |
| 78 | 289.97 ± 45.55 | 90.99 ± 23.03 | 70.66 ± 25.62 | 33.25 ± 2.89 | 24.47 ± 4.16 | 11.12 ± 1.93 | 59.48 ± 12.81 |
| 79 | 235.21 ± 39.52 | 163.07 ± 29.39 | 0.00 ± 0.00 | 5.61 ± 2.20 | 16.78 ± 1.14 | 0.55 ± 0.68 | 49.20 ± 15.13 |
| 79 | 223.45 ± 54.51 | 145.38 ± 35.76 | 0.00 ± 0.00 | 5.60 ± 2.59 | 16.62 ± 1.14 | 0.56 ± 0.69 | 55.30 ± 17.25 |
| 79 | 321.03 ± 37.33 | 223.44 ± 29.62 | 0.00 ± 0.00 | 6.06 ± 2.20 | 17.04 ± 1.17 | 0.76 ± 0.74 | 73.74 ± 14.74 |
| 88 | 56.97 ± 11.68 | 53.94 ± 10.86 | 0.00 ± 0.00 | 0.02 ± 0.20 | 0.00 ± 0.04 | 0.00 ± 0.00 | 3.01 ± 1.14 |
| 88 | 53.55 ± 13.39 | 50.79 ± 12.22 | 0.00 ± 0.00 | 0.06 ± 0.32 | 0.00 ± 0.00 | 0.00 ± 0.00 | 2.69 ± 1.36 |
| 88 | 53.06 ± 10.99 | 50.28 ± 9.91 | 0.00 ± 0.00 | 0.04 ± 0.25 | 0.00 ± 0.00 | 0.00 ± 0.00 | 2.75 ± 1.31 |
| 96 | 374.83 ± 53.03 | 229.15 ± 43.23 | 0.00 ± 0.00 | 44.88 ± 7.64 | 7.18 ± 3.56 | 62.31 ± 5.99 | 31.32 ± 6.78 |
| 96 | 395.80 ± 56.05 | 246.99 ± 47.40 | 0.00 ± 0.00 | 48.65 ± 7.79 | 7.27 ± 3.41 | 64.82 ± 6.07 | 28.06 ± 7.72 |
| 96 | 401.77 ± 48.04 | 260.95 ± 42.67 | 0.00 ± 0.00 | 47.91 ± 6.74 | 7.62 ± 3.16 | 63.93 ± 5.29 | 21.37 ± 7.62 |
| 97 | 114.39 ± 19.13 | 21.23 ± 6.15 | 0.00 ± 0.00 | 37.76 ± 5.09 | 24.56 ± 4.90 | 14.37 ± 2.67 | 16.47 ± 5.57 |
| 97 | 105.81 ± 19.07 | 19.25 ± 5.58 | 0.00 ± 0.00 | 35.69 ± 5.40 | 22.68 ± 4.52 | 13.15 ± 2.87 | 15.05 ± 4.65 |
| 97 | 106.46 ± 18.18 | 19.95 ± 5.68 | 0.00 ± 0.00 | 34.56 ± 5.27 | 22.61 ± 4.45 | 12.83 ± 3.20 | 16.51 ± 5.13 |
| 98 | 106.85 ± 26.77 | 30.96 ± 10.25 | 0.00 ± 0.00 | 30.49 ± 7.91 | 36.97 ± 6.27 | 8.43 ± 6.79 | 42.86 ± 9.61 |
| 98 | 102.85 ± 32.86 | 29.99 ± 13.20 | 0.00 ± 0.00 | 29.57 ± 8.90 | 36.05 ± 7.58 | 7.24 ± 5.16 | 41.19 ± 10.14 |
| 98 | 115.13 ± 37.90 | 35.65 ± 16.09 | 0.00 ± 0.00 | 34.38 ± 10.04 | 37.47 ± 7.52 | 7.64 ± 6.79 | 45.33 ± 11.51 |
| 99 | 49.73 ± 51.84 | 25.16 ± 31.43 | 0.00 ± 0.00 | 5.47 ± 8.21 | 17.41 ± 9.26 | 1.70 ± 4.25 | 14.65 ± 10.43 |
| 99 | 46.10 ± 31.07 | 23.99 ± 18.14 | 0.00 ± 0.00 | 4.83 ± 5.54 | 16.83 ± 7.51 | 0.46 ± 1.15 | 13.53 ± 7.05 |
| 99 | 45.49 ± 31.14 | 23.61 ± 16.64 | 0.00 ± 0.00 | 4.39 ± 5.25 | 16.80 ± 8.37 | 0.69 ± 2.31 | 13.21 ± 7.34 |
| 105 | 23.10 ± 30.05 | 13.44 ± 18.57 | 0.00 ± 0.00 | 0.00 ± 0.00 | 0.00 ± 0.00 | 0.00 ± 0.00 | 9.65 ± 12.27 |
| 105 | 31.53 ± 33.18 | 18.47 ± 20.70 | 0.00 ± 0.00 | 0.00 ± 0.00 | 0.00 ± 0.00 | 0.01 ± 0.11 | 13.05 ± 13.30 |
| 105 | 58.07 ± 29.61 | 38.08 ± 20.68 | 0.00 ± 0.00 | 0.00 ± 0.00 | 0.00 ± 0.00 | 0.00 ± 0.00 | 20.00 ± 10.60 |
| 107 | 112.30 ± 82.09 | 101.06 ± 70.10 | 0.00 ± 0.00 | 3.68 ± 5.80 | 0.19 ± 0.82 | 1.03 ± 2.35 | 6.34 ± 6.08 |
| 107 | 124.89 ± 85.89 | 108.21 ± 70.29 | 0.00 ± 0.00 | 6.87 ± 7.38 | 0.51 ± 1.40 | 2.04 ± 2.96 | 7.25 ± 6.20 |
| 107 | 297.89 ± 62.14 | 254.64 ± 50.78 | 0.00 ± 0.00 | 17.34 ± 8.04 | 0.88 ± 1.57 | 5.67 ± 3.79 | 19.34 ± 5.01 |

| | | | | | | | |
|---|---|---|---|---|---|---|---|
| 108 | 79.22 ± 129.78 | 23.13 ± 43.97 | 0.00 ± 0.00 | 6.26 ± 11.93 | 7.91 ± 12.21 | 11.98 ± 18.91 | 29.95 ± 49.39 |
| 108 | 257.94 ± 122.90 | 52.45 ± 36.62 | 0.00 ± 0.00 | 24.93 ± 14.03 | 21.53 ± 13.32 | 54.23 ± 19.63 | 104.81 ± 50.39 |
| 108 | 172.44 ± 74.85 | 21.30 ± 24.18 | 0.00 ± 0.00 | 16.59 ± 8.27 | 26.57 ± 9.91 | 40.47 ± 15.11 | 67.50 ± 33.49 |
| 109 | 98.38 ± 101.30 | 0.00 ± 0.00 | 0.00 ± 0.00 | 15.86 ± 19.82 | 12.19 ± 18.90 | 15.34 ± 16.36 | 54.99 ± 60.38 |
| 109 | 266.34 ± 61.45 | 0.00 ± 0.00 | 0.00 ± 0.00 | 58.52 ± 11.39 | 39.24 ± 18.17 | 19.65 ± 4.92 | 148.93 ± 37.13 |
| 109 | 228.79 ± 43.20 | 0.00 ± 0.00 | 0.00 ± 0.00 | 52.41 ± 13.89 | 36.31 ± 11.52 | 19.42 ± 6.89 | 120.66 ± 21.60 |
| 110 | 105.89 ± 110.32 | 21.76 ± 27.04 | 0.00 ± 0.00 | 15.44 ± 17.36 | 11.63 ± 12.96 | 7.32 ± 8.78 | 49.74 ± 58.83 |
| 110 | 200.01 ± 63.72 | 51.57 ± 23.26 | 0.00 ± 0.00 | 16.13 ± 6.18 | 41.92 ± 7.18 | 16.65 ± 6.36 | 73.74 ± 40.22 |
| 110 | 132.85 ± 62.42 | 32.44 ± 16.20 | 0.00 ± 0.00 | 13.31 ± 6.63 | 36.91 ± 15.46 | 10.30 ± 5.85 | 39.89 ± 23.38 |
| 111 | 123.43 ± 118.29 | 30.79 ± 31.03 | 0.00 ± 0.00 | 11.11 ± 12.59 | 19.56 ± 16.99 | 13.43 ± 20.18 | 48.54 ± 46.90 |
| 111 | 277.44 ± 74.44 | 121.40 ± 40.40 | 0.00 ± 0.00 | 24.34 ± 8.68 | 37.13 ± 8.53 | 8.50 ± 4.13 | 86.08 ± 23.02 |
| 111 | 187.35 ± 118.62 | 61.41 ± 48.39 | 0.00 ± 0.00 | 13.01 ± 9.34 | 30.66 ± 13.66 | 6.35 ± 8.82 | 75.92 ± 48.01 |
| 112 | 117.47 ± 114.62 | 0.00 ± 0.00 | 0.00 ± 0.00 | 17.39 ± 20.65 | 19.67 ± 20.47 | 7.88 ± 10.50 | 72.52 ± 65.88 |
| 112 | 43.50 ± 20.98 | 0.00 ± 0.00 | 0.00 ± 0.00 | 4.57 ± 3.64 | 12.33 ± 5.46 | 0.40 ± 1.00 | 26.20 ± 12.90 |
| 112 | 100.10 ± 46.03 | 0.00 ± 0.00 | 0.00 ± 0.00 | 9.14 ± 7.47 | 17.90 ± 9.55 | 2.80 ± 2.59 | 70.27 ± 29.88 |
| 114 | 95.73 ± 100.42 | 56.07 ± 70.14 | 0.00 ± 0.00 | 2.65 ± 4.87 | 2.00 ± 3.71 | 0.36 ± 0.85 | 34.65 ± 24.17 |
| 114 | 23.74 ± 14.04 | 9.02 ± 7.36 | 0.00 ± 0.00 | 0.00 ± 0.00 | 0.00 ± 0.00 | 0.00 ± 0.00 | 14.72 ± 7.66 |
| 114 | 139.40 ± 57.40 | 83.93 ± 39.15 | 0.00 ± 0.00 | 0.02 ± 0.17 | 0.00 ± 0.00 | 0.00 ± 0.00 | 55.45 ± 18.94 |
| 129 | 46.07 ± 54.53 | 23.61 ± 25.91 | 0.00 ± 0.00 | 5.13 ± 7.19 | 4.99 ± 8.03 | 6.04 ± 8.76 | 6.30 ± 7.35 |
| 129 | 58.28 ± 51.66 | 30.27 ± 24.63 | 0.00 ± 0.00 | 6.50 ± 6.96 | 5.42 ± 6.14 | 8.63 ± 9.64 | 7.47 ± 6.71 |
| 129 | 46.68 ± 74.08 | 22.58 ± 33.58 | 0.00 ± 0.00 | 5.73 ± 10.21 | 5.00 ± 9.51 | 7.07 ± 13.16 | 6.30 ± 9.78 |
| 150 | 251.68 ± 202.99 | 40.80 ± 43.40 | 149.32 ± 115.99 | 8.11 ± 9.08 | 1.25 ± 2.12 | 24.74 ± 23.67 | 27.48 ± 29.37 |
| 150 | 348.86 ± 192.79 | 54.40 ± 45.31 | 211.41 ± 106.25 | 10.47 ± 8.04 | 1.30 ± 1.98 | 33.65 ± 20.45 | 37.62 ± 27.97 |
| 150 | 224.62 ± 207.47 | 33.02 ± 44.70 | 140.91 ± 117.40 | 6.83 ± 9.47 | 1.78 ± 3.34 | 19.29 ± 23.22 | 22.79 ± 30.12 |
| 151 | 147.98 ± 134.26 | 51.64 ± 46.49 | 0.00 ± 0.00 | 35.84 ± 32.62 | 16.97 ± 16.17 | 24.87 ± 24.55 | 18.67 ± 18.79 |
| 151 | 208.45 ± 122.79 | 72.98 ± 43.30 | 0.00 ± 0.00 | 49.80 ± 29.32 | 23.21 ± 15.05 | 34.88 ± 22.21 | 27.56 ± 19.31 |
| 151 | 109.34 ± 122.75 | 37.38 ± 41.80 | 0.00 ± 0.00 | 26.15 ± 30.04 | 13.43 ± 15.64 | 17.84 ± 23.16 | 14.55 ± 17.68 |
| 168 | 17.10 ± 60.90 | 8.19 ± 30.19 | 0.00 ± 0.00 | 3.63 ± 12.78 | 1.68 ± 6.25 | 2.25 ± 8.51 | 1.36 ± 4.86 |
| 168 | 76.89 ± 100.36 | 45.08 ± 57.59 | 0.00 ± 0.00 | 12.11 ± 17.91 | 5.24 ± 8.94 | 7.65 ± 13.81 | 6.81 ± 9.22 |
| 168 | 11.24 ± 50.28 | 5.73 ± 27.27 | 0.00 ± 0.00 | 2.13 ± 9.24 | 1.01 ± 5.14 | 1.42 ± 6.25 | 0.95 ± 4.39 |

**Table C: List of residues with the most contacts in the parallel binding mode. Calculated from the three replicas of the 'Gathering' simulations. 'Resid' column gives the number of the residue, 'All' stands for the number of all contacts, 'Hydrophob.' corresponds to contacts to hydrophobic parts of the protein, 'Charged' refers to contacts to the charged part of the proteins, 'CAHA' is for contacts to the Cα and Hα atoms of the protein backbone, 'NH' provides data for contacts to the N and H atoms of the protein backbone, 'OC' describes contacts to the O and C atoms of the protein backbone, and 'other' stands for contacts to all the remaining part of the protein residues. The values are numbers of contacts and the errors are standard deviations.**

| Resid | All | Hydrophob. | Charged | CAHA | NH | OC | Other |
|---|---|---|---|---|---|---|---|
| 20 | 4.07 ± 22.66 | 0.49 ± 3.31 | 0.00 ± 0.00 | 0.75 ± 4.41 | 0.46 ± 2.61 | 0.87 ± 4.69 | 1.50 ± 8.15 |
| 20 | 0.00 ± 0.00 | 0.00 ± 0.00 | 0.00 ± 0.00 | 0.00 ± 0.00 | 0.00 ± 0.00 | 0.00 ± 0.00 | 0.00 ± 0.00 |
| 20 | 86.93 ± 85.50 | 17.36 ± 22.73 | 0.00 ± 0.00 | 19.26 ± 20.02 | 8.74 ± 9.34 | 12.31 ± 13.10 | 25.98 ± 27.51 |
| 21 | 63.61 ± 59.28 | 15.20 ± 21.24 | 0.00 ± 0.00 | 0.81 ± 3.30 | 0.80 ± 4.09 | 0.30 ± 1.78 | 46.50 ± 36.77 |
| 21 | 0.06 ± 0.77 | 0.00 ± 0.00 | 0.00 ± 0.00 | 0.00 ± 0.00 | 0.00 ± 0.00 | 0.00 ± 0.00 | 0.06 ± 0.77 |
| 21 | 230.36 ± 180.36 | 85.42 ± 72.54 | 0.00 ± 0.00 | 14.30 ± 13.10 | 22.67 ± 21.26 | 13.32 ± 11.51 | 87.44 ± 69.77 |
| 23 | 13.20 ± 12.48 | 4.42 ± 4.47 | 0.00 ± 0.00 | 0.13 ± 0.62 | 0.08 ± 0.64 | 0.02 ± 0.21 | 8.55 ± 8.68 |
| 23 | 1.10 ± 2.77 | 0.12 ± 0.75 | 0.00 ± 0.00 | 0.00 ± 0.00 | 0.00 ± 0.00 | 0.00 ± 0.00 | 0.97 ± 2.52 |
| 23 | 65.62 ± 42.55 | 17.62 ± 12.15 | 0.00 ± 0.00 | 3.28 ± 2.85 | 5.14 ± 4.42 | 0.47 ± 1.03 | 38.93 ± 24.43 |
| 39 | 29.84 ± 15.98 | 2.70 ± 4.07 | 0.00 ± 0.00 | 2.12 ± 3.76 | 0.20 ± 0.92 | 24.48 ± 8.61 | 0.35 ± 1.60 |

| | | | | | | | |
|---|---|---|---|---|---|---|---|
| 39 | 9.00 ± 9.01 | 0.30 ± 1.28 | 0.00 ± 0.00 | 0.75 ± 1.97 | 0.11 ± 0.46 | 7.82 ± 6.13 | 0.03 ± 0.34 |
| 39 | 86.82 ± 55.10 | 15.42 ± 13.32 | 0.00 ± 0.00 | 12.67 ± 9.97 | 1.05 ± 1.61 | 37.40 ± 16.09 | 18.29 ± 19.72 |
| 40 | 155.75 ± 32.98 | 0.00 ± 0.00 | 0.00 ± 0.00 | 23.66 ± 5.68 | 18.64 ± 6.35 | 62.82 ± 11.28 | 50.62 ± 11.77 |
| 40 | 114.83 ± 28.00 | 0.00 ± 0.00 | 0.00 ± 0.00 | 15.11 ± 4.56 | 11.81 ± 5.35 | 53.19 ± 9.01 | 34.71 ± 10.48 |
| 40 | 155.09 ± 35.29 | 0.00 ± 0.00 | 0.00 ± 0.00 | 23.58 ± 6.22 | 23.82 ± 9.74 | 58.33 ± 10.10 | 50.58 ± 12.48 |
| 41 | 522.98 ± 59.58 | 169.87 ± 28.39 | 166.12 ± 51.37 | 64.29 ± 11.85 | 32.33 ± 7.52 | 18.14 ± 3.82 | 72.23 ± 18.96 |
| 41 | 528.59 ± 71.78 | 139.85 ± 31.95 | 228.44 ± 48.17 | 43.76 ± 5.29 | 23.04 ± 5.79 | 13.02 ± 3.00 | 80.48 ± 19.09 |
| 41 | 534.26 ± 79.96 | 164.24 ± 26.16 | 193.31 ± 58.75 | 59.20 ± 11.48 | 34.32 ± 9.77 | 18.02 ± 4.04 | 65.19 ± 17.85 |
| 42 | 388.53 ± 44.14 | 263.99 ± 26.18 | 0.00 ± 0.00 | 23.78 ± 8.05 | 51.33 ± 9.68 | 33.26 ± 14.26 | 16.17 ± 9.32 |
| 42 | 331.69 ± 32.29 | 249.81 ± 24.03 | 0.00 ± 0.00 | 11.75 ± 2.25 | 30.44 ± 4.99 | 5.21 ± 2.73 | 34.48 ± 7.49 |
| 42 | 382.39 ± 48.65 | 269.11 ± 36.37 | 0.00 ± 0.00 | 17.82 ± 6.35 | 47.21 ± 10.32 | 21.94 ± 13.29 | 24.92 ± 11.64 |
| 78 | 206.96 ± 46.59 | 21.40 ± 8.16 | 147.14 ± 35.59 | 1.83 ± 1.70 | 0.01 ± 0.08 | 0.01 ± 0.09 | 36.58 ± 8.92 |
| 78 | 236.93 ± 56.57 | 30.00 ± 15.88 | 162.65 ± 31.68 | 5.69 ± 2.80 | 0.31 ± 1.17 | 0.13 ± 0.39 | 38.15 ± 15.27 |
| 78 | 162.06 ± 38.49 | 18.03 ± 8.92 | 117.59 ± 29.97 | 2.60 ± 2.09 | 0.03 ± 0.18 | 0.02 ± 0.15 | 26.05 ± 9.01 |
| 79 | 17.11 ± 37.90 | 13.79 ± 31.91 | 0.00 ± 0.00 | 0.04 ± 0.27 | 1.07 ± 1.25 | 0.01 ± 0.11 | 2.19 ± 5.35 |
| 79 | 97.67 ± 40.13 | 80.34 ± 33.81 | 0.00 ± 0.00 | 0.18 ± 0.55 | 3.77 ± 2.13 | 0.14 ± 0.35 | 13.24 ± 6.36 |
| 79 | 76.74 ± 56.64 | 61.52 ± 48.02 | 0.00 ± 0.00 | 0.17 ± 0.52 | 2.17 ± 1.97 | 0.01 ± 0.12 | 10.04 ± 8.41 |
| 107 | 55.04 ± 95.97 | 45.54 ± 79.96 | 0.00 ± 0.00 | 3.06 ± 6.66 | 0.48 ± 1.55 | 2.96 ± 4.29 | 2.99 ± 6.14 |
| 107 | 181.38 ± 76.11 | 163.07 ± 63.39 | 0.00 ± 0.00 | 4.57 ± 5.91 | 0.24 ± 1.14 | 1.78 ± 3.43 | 11.72 ± 6.09 |
| 107 | 128.97 ± 99.31 | 111.63 ± 88.18 | 0.00 ± 0.00 | 3.39 ± 5.48 | 0.20 ± 0.71 | 1.42 ± 3.35 | 7.54 ± 6.53 |
| 108 | 315.04 ± 87.41 | 89.01 ± 37.09 | 0.00 ± 0.00 | 33.07 ± 11.65 | 15.13 ± 9.79 | 58.19 ± 16.50 | 119.64 ± 36.43 |
| 108 | 70.12 ± 103.15 | 13.09 ± 31.65 | 0.00 ± 0.00 | 5.26 ± 9.57 | 9.18 ± 11.88 | 15.63 ± 18.45 | 26.95 ± 40.78 |
| 108 | 61.90 ± 105.84 | 16.68 ± 38.92 | 0.00 ± 0.00 | 4.97 ± 9.34 | 6.25 ± 9.97 | 10.24 ± 14.31 | 21.90 ± 37.83 |
| 109 | 305.97 ± 47.33 | 0.00 ± 0.00 | 0.00 ± 0.00 | 68.96 ± 14.28 | 46.99 ± 11.67 | 37.56 ± 15.47 | 152.46 ± 27.36 |
| 109 | 238.08 ± 50.83 | 0.00 ± 0.00 | 0.00 ± 0.00 | 33.07 ± 11.87 | 24.63 ± 15.04 | 54.87 ± 16.98 | 125.51 ± 29.06 |
| 109 | 219.21 ± 86.23 | 0.00 ± 0.00 | 0.00 ± 0.00 | 30.03 ± 14.71 | 23.97 ± 17.67 | 57.85 ± 15.22 | 104.83 ± 48.64 |
| 110 | 187.92 ± 31.17 | 55.29 ± 11.60 | 0.00 ± 0.00 | 27.35 ± 13.68 | 54.24 ± 7.94 | 18.72 ± 6.27 | 32.32 ± 17.81 |
| 110 | 201.54 ± 77.11 | 35.53 ± 14.87 | 0.00 ± 0.00 | 48.59 ± 17.70 | 37.24 ± 9.41 | 30.21 ± 12.62 | 49.97 ± 46.81 |
| 110 | 271.26 ± 71.66 | 42.97 ± 17.67 | 0.00 ± 0.00 | 56.08 ± 10.15 | 36.63 ± 11.29 | 33.76 ± 8.83 | 101.60 ± 54.66 |
| 111 | 229.13 ± 36.43 | 52.57 ± 15.29 | 0.00 ± 0.00 | 22.19 ± 5.01 | 44.80 ± 5.43 | 24.71 ± 8.03 | 84.85 ± 16.52 |
| 111 | 198.11 ± 60.45 | 50.09 ± 24.32 | 0.00 ± 0.00 | 20.24 ± 8.53 | 43.02 ± 11.74 | 10.12 ± 4.01 | 74.64 ± 26.42 |
| 111 | 225.01 ± 43.98 | 54.32 ± 23.06 | 0.00 ± 0.00 | 23.96 ± 6.54 | 49.73 ± 7.34 | 15.90 ± 8.54 | 82.20 ± 19.85 |
| 112 | 285.93 ± 53.20 | 0.00 ± 0.00 | 0.00 ± 0.00 | 52.02 ± 16.22 | 46.26 ± 9.98 | 18.53 ± 8.69 | 169.12 ± 21.74 |
| 112 | 185.84 ± 34.38 | 0.00 ± 0.00 | 0.00 ± 0.00 | 19.18 ± 5.30 | 30.36 ± 7.97 | 6.81 ± 1.96 | 129.49 ± 21.97 |
| 112 | 229.13 ± 37.00 | 0.00 ± 0.00 | 0.00 ± 0.00 | 29.19 ± 12.24 | 35.16 ± 7.17 | 10.61 ± 5.18 | 154.54 ± 19.58 |
| 113 | 125.18 ± 82.66 | 40.25 ± 30.39 | 0.00 ± 0.00 | 9.69 ± 7.82 | 34.10 ± 15.86 | 19.23 ± 10.57 | 21.91 ± 24.37 |
| 113 | 11.31 ± 8.86 | 0.63 ± 1.78 | 0.00 ± 0.00 | 0.16 ± 0.43 | 5.50 ± 3.84 | 4.67 ± 3.07 | 0.35 ± 2.97 |
| 113 | 48.33 ± 54.08 | 12.33 ± 20.37 | 0.00 ± 0.00 | 2.93 ± 5.01 | 16.73 ± 12.76 | 11.57 ± 6.94 | 4.77 ± 12.91 |
| 114 | 75.96 ± 59.71 | 39.97 ± 40.84 | 0.00 ± 0.00 | 2.59 ± 2.08 | 2.40 ± 2.53 | 0.00 ± 0.06 | 31.00 ± 21.73 |
| 114 | 180.87 ± 28.88 | 111.30 ± 23.32 | 0.00 ± 0.00 | 0.99 ± 0.95 | 0.00 ± 0.06 | 0.02 ± 0.17 | 68.55 ± 6.49 |
| 114 | 146.44 ± 82.70 | 87.56 ± 56.36 | 0.00 ± 0.00 | 1.88 ± 1.75 | 0.73 ± 1.74 | 0.01 ± 0.19 | 52.04 ± 28.74 |
| 129 | 0.04 ± 0.74 | 0.03 ± 0.54 | 0.00 ± 0.00 | 0.00 ± 0.00 | 0.00 ± 0.00 | 0.00 ± 0.09 | 0.01 ± 0.15 |
| 129 | 62.98 ± 65.12 | 32.06 ± 31.66 | 0.00 ± 0.00 | 6.86 ± 8.64 | 7.57 ± 9.60 | 7.24 ± 8.62 | 9.25 ± 10.32 |
| 129 | 0.00 ± 0.00 | 0.00 ± 0.00 | 0.00 ± 0.00 | 0.00 ± 0.00 | 0.00 ± 0.00 | 0.00 ± 0.00 | 0.00 ± 0.00 |
| 146 | 78.00 ± 41.88 | 45.10 ± 23.38 | 0.00 ± 0.00 | 5.89 ± 4.90 | 1.04 ± 1.95 | 0.43 ± 0.86 | 25.54 ± 15.97 |
| 146 | 0.15 ± 1.28 | 0.14 ± 1.17 | 0.00 ± 0.00 | 0.00 ± 0.06 | 0.00 ± 0.00 | 0.00 ± 0.00 | 0.01 ± 0.13 |
| 146 | 18.39 ± 22.75 | 8.80 ± 14.71 | 0.00 ± 0.00 | 0.46 ± 1.73 | 0.61 ± 1.29 | 0.18 ± 1.04 | 10.79 ± 11.20 |
| 150 | 2.21 ± 18.64 | 0.48 ± 5.17 | 0.58 ± 2.88 | 0.28 ± 2.97 | 0.08 ± 0.90 | 0.58 ± 5.41 | 0.21 ± 2.32 |
| 150 | 263.60 ± 166.53 | 45.22 ± 41.60 | 131.91 ± 98.09 | 15.64 ± 10.27 | 2.47 ± 3.08 | 44.01 ± 21.94 | 24.35 ± 25.50 |
| 150 | 0.73 ± 9.24 | 0.05 ± 1.16 | 0.45 ± 4.92 | 0.04 ± 0.73 | 0.01 ± 0.13 | 0.11 ± 1.30 | 0.05 ± 1.03 |
| 151 | 3.02 ± 22.78 | 0.62 ± 5.42 | 0.00 ± 0.00 | 0.74 ± 6.35 | 0.32 ± 2.98 | 1.16 ± 7.54 | 0.17 ± 1.74 |
| 151 | 214.93 ± 108.26 | 66.07 ± 38.17 | 0.00 ± 0.00 | 54.30 ± 26.65 | 25.87 ± 14.19 | 43.43 ± 22.11 | 25.27 ± 16.99 |
| 151 | 4.57 ± 22.30 | 0.93 ± 5.24 | 0.00 ± 0.00 | 0.57 ± 3.17 | 0.19 ± 1.16 | 1.89 ± 7.99 | 0.86 ± 4.93 |
| 152 | 27.30 ± 29.90 | 0.00 ± 0.00 | 0.00 ± 0.00 | 2.20 ± 4.73 | 1.15 ± 6.42 | 17.82 ± 11.96 | 6.13 ± 10.51 |
| 152 | 87.18 ± 65.64 | 0.00 ± 0.00 | 0.00 ± 0.00 | 12.92 ± 9.89 | 28.61 ± 17.93 | 18.44 ± 23.43 | 27.20 ± 18.49 |
| 152 | 15.05 ± 37.02 | 0.00 ± 0.00 | 0.00 ± 0.00 | 2.01 ± 5.25 | 1.74 ± 5.47 | 7.28 ± 15.67 | 4.15 ± 10.85 |

| | | | | | | | |
|---|---|---|---|---|---|---|---|
| 153 | 80.58 ± 45.14 | 22.21 ± 13.40 | 0.00 ± 0.00 | 21.34 ± 11.87 | 6.20 ± 5.05 | 11.55 ± 6.75 | 19.28 ± 10.76 |
| 153 | 78.39 ± 123.94 | 23.51 ± 37.70 | 0.00 ± 0.00 | 14.17 ± 24.68 | 14.96 ± 22.16 | 4.38 ± 8.52 | 21.37 ± 39.83 |
| 153 | 32.75 ± 70.77 | 11.34 ± 24.07 | 0.00 ± 0.00 | 7.96 ± 16.03 | 4.15 ± 9.66 | 4.06 ± 8.87 | 7.56 ± 15.62 |
| 154 | 635.59 ± 247.98 | 144.07 ± 63.90 | 282.73 ± 101.74 | 24.74 ± 12.44 | 34.48 ± 17.35 | 40.31 ± 18.45 | 109.27 ± 44.96 |
| 154 | 119.60 ± 201.51 | 12.94 ± 32.66 | 76.43 ± 122.85 | 1.90 ± 7.18 | 7.49 ± 14.94 | 2.36 ± 9.70 | 18.48 ± 32.49 |
| 154 | 206.76 ± 225.50 | 35.15 ± 61.58 | 129.98 ± 97.92 | 5.54 ± 11.87 | 10.35 ± 20.33 | 9.49 ± 18.84 | 29.86 ± 42.96 |
| 155 | 109.62 ± 53.44 | 48.69 ± 26.69 | 0.00 ± 0.00 | 22.70 ± 11.66 | 18.70 ± 9.76 | 10.15 ± 7.03 | 9.38 ± 5.69 |
| 155 | 6.96 ± 36.91 | 4.31 ± 23.98 | 0.00 ± 0.00 | 0.80 ± 4.69 | 0.53 ± 2.84 | 0.15 ± 1.46 | 1.17 ± 4.89 |
| 155 | 28.38 ± 73.04 | 17.79 ± 47.25 | 0.00 ± 0.00 | 3.98 ± 9.40 | 2.47 ± 5.77 | 1.00 ± 3.09 | 3.97 ± 10.29 |
| 156 | 152.04 ± 76.14 | 131.52 ± 64.44 | 0.00 ± 0.00 | 8.20 ± 6.14 | 11.24 ± 8.15 | 1.08 ± 2.89 | 9.57 ± 7.38 |
| 156 | 1.40 ± 13.09 | 0.96 ± 8.53 | 0.00 ± 0.00 | 0.08 ± 0.98 | 0.29 ± 2.75 | 0.07 ± 1.14 | 0.25 ± 2.45 |
| 156 | 19.55 ± 58.81 | 18.41 ± 51.39 | 0.00 ± 0.00 | 0.87 ± 2.86 | 2.12 ± 6.34 | 0.62 ± 2.58 | 1.92 ± 5.95 |

**Table D: List of residues with the most contacts in the upright binding mode. Calculated from the three replicas of the 'Gathering' simulations. 'Resid' column gives the number of the residue, 'All' stands for the number of all contacts, 'Hydrophob.' means contacts to hydrophobic parts of the protein, 'Charged' means contacts to the charged part of the proteins, 'CAHA' refers to contacts to the Cα and Hα atoms of the protein backbone, 'NH' corresponds to contacts to the N and H atoms of the protein backbone, 'OC' is for contacts to the O and C atoms of the protein backbone, and 'other' provides data for contacts to all the remaining part of the protein residues. The values are numbers of contacts and the errors are standard deviations.**

| Resid | All | Hydrophob. | Charged | CAHA | NH | OC | Other |
|---|---|---|---|---|---|---|---|
| 20 | 160.17 ± 100.12 | 85.48 ± 54.62 | 0.00 ± 0.00 | 20.44 ± 14.71 | 11.52 ± 8.38 | 8.35 ± 9.49 | 34.62 ± 25.20 |
| 20 | 31.27 ± 41.64 | 18.64 ± 19.91 | 0.00 ± 0.00 | 3.47 ± 6.07 | 1.55 ± 3.67 | 1.58 ± 3.67 | 4.64 ± 11.15 |
| 20 | 54.16 ± 93.01 | 19.02 ± 40.21 | 0.00 ± 0.00 | 4.43 ± 10.98 | 2.69 ± 6.57 | 2.11 ± 5.82 | 8.32 ± 20.12 |
| 21 | 15.42 ± 28.07 | 1.63 ± 8.00 | 0.00 ± 0.00 | 1.53 ± 4.83 | 4.97 ± 6.80 | 5.16 ± 5.66 | 0.79 ± 6.28 |
| 21 | 3.31 ± 6.58 | 0.03 ± 0.43 | 0.00 ± 0.00 | 0.18 ± 0.75 | 1.41 ± 3.04 | 1.01 ± 2.25 | 0.01 ± 0.18 |
| 21 | 3.99 ± 15.24 | 0.35 ± 3.05 | 0.00 ± 0.00 | 0.22 ± 1.36 | 1.05 ± 3.62 | 0.61 ± 2.41 | 0.44 ± 6.87 |
| 22 | 85.62 ± 57.27 | 68.00 ± 49.40 | 0.00 ± 0.00 | 4.82 ± 3.99 | 1.33 ± 2.98 | 0.11 ± 0.38 | 4.76 ± 3.93 |
| 22 | 19.95 ± 21.17 | 15.39 ± 17.41 | 0.00 ± 0.00 | 0.29 ± 1.10 | 0.12 ± 0.89 | 0.00 ± 0.04 | 0.64 ± 1.81 |
| 22 | 17.17 ± 33.47 | 10.27 ± 25.66 | 0.00 ± 0.00 | 0.44 ± 1.56 | 0.14 ± 1.22 | 0.00 ± 0.04 | 0.66 ± 1.92 |
| 38 | 144.67 ± 35.50 | 39.57 ± 15.47 | 65.50 ± 17.07 | 0.79 ± 1.45 | 0.03 ± 0.21 | 2.84 ± 3.00 | 35.17 ± 8.41 |
| 38 | 34.76 ± 53.09 | 4.33 ± 11.50 | 14.97 ± 21.82 | 0.04 ± 0.21 | 0.00 ± 0.00 | 0.44 ± 1.47 | 5.42 ± 11.41 |
| 38 | 44.65 ± 63.72 | 6.75 ± 14.46 | 14.87 ± 26.97 | 0.07 ± 0.34 | 0.00 ± 0.00 | 0.70 ± 1.67 | 7.52 ± 14.25 |
| 39 | 62.19 ± 29.02 | 15.98 ± 8.82 | 0.00 ± 0.00 | 1.92 ± 2.93 | 1.96 ± 2.76 | 1.25 ± 2.43 | 38.69 ± 15.66 |
| 39 | 14.74 ± 23.42 | 2.46 ± 6.06 | 0.00 ± 0.00 | 0.33 ± 1.14 | 0.24 ± 0.85 | 0.32 ± 1.13 | 6.82 ± 12.19 |
| 39 | 25.54 ± 39.66 | 4.69 ± 9.34 | 0.00 ± 0.00 | 0.76 ± 1.95 | 0.45 ± 1.16 | 0.65 ± 1.84 | 10.56 ± 21.51 |
| 41 | 443.25 ± 64.71 | 155.33 ± 27.45 | 146.04 ± 31.43 | 30.71 ± 9.51 | 5.57 ± 3.14 | 21.81 ± 6.14 | 83.65 ± 19.61 |
| 41 | 165.55 ± 140.96 | 31.20 ± 51.08 | 101.24 ± 64.26 | 3.80 ± 8.39 | 0.37 ± 1.11 | 2.57 ± 5.57 | 18.78 ± 22.20 |
| 41 | 156.90 ± 184.66 | 38.13 ± 64.59 | 51.61 ± 58.43 | 5.98 ± 10.65 | 0.62 ± 1.52 | 4.09 ± 7.77 | 20.28 ± 33.06 |
| 42 | 412.11 ± 82.53 | 239.74 ± 51.78 | 0.00 ± 0.00 | 29.20 ± 6.03 | 49.24 ± 8.19 | 62.79 ± 9.19 | 33.49 ± 13.44 |
| 42 | 265.18 ± 145.36 | 191.23 ± 75.13 | 0.00 ± 0.00 | 5.19 ± 7.67 | 9.29 ± 15.71 | 17.07 ± 17.63 | 43.41 ± 17.96 |
| 42 | 323.95 ± 131.07 | 226.76 ± 66.51 | 0.00 ± 0.00 | 7.32 ± 10.62 | 14.09 ± 18.75 | 19.25 ± 22.57 | 48.85 ± 14.13 |
| 43 | 226.00 ± 35.17 | 0.00 ± 0.00 | 0.00 ± 0.00 | 52.74 ± 8.61 | 23.08 ± 5.13 | 32.56 ± 10.32 | 116.36 ± 18.20 |
| 43 | 65.53 ± 85.88 | 0.00 ± 0.00 | 0.00 ± 0.00 | 13.86 ± 17.97 | 3.98 ± 7.40 | 7.12 ± 13.50 | 20.86 ± 37.04 |
| 43 | 81.42 ± 109.89 | 0.00 ± 0.00 | 0.00 ± 0.00 | 14.61 ± 22.45 | 5.13 ± 9.41 | 8.72 ± 15.33 | 27.31 ± 50.53 |
| 57 | 0.00 ± 0.00 | 0.00 ± 0.00 | 0.00 ± 0.00 | 0.00 ± 0.00 | 0.00 ± 0.00 | 0.00 ± 0.00 | 0.00 ± 0.00 |
| 57 | 100.95 ± 147.12 | 30.51 ± 31.89 | 0.00 ± 0.00 | 12.89 ± 16.86 | 3.24 ± 4.46 | 13.41 ± 19.95 | 85.92 ± 81.13 |
| 57 | 0.00 ± 0.00 | 0.00 ± 0.00 | 0.00 ± 0.00 | 0.00 ± 0.00 | 0.00 ± 0.00 | 0.00 ± 0.00 | 0.00 ± 0.00 |
| 78 | 198.88 ± 66.39 | 22.89 ± 22.75 | 129.27 ± 34.66 | 11.54 ± 7.14 | 5.76 ± 4.15 | 2.44 ± 3.59 | 20.70 ± 11.49 |

| | | | | | | | |
|---|---|---|---|---|---|---|---|
| 78 | 130.58 ± 88.33 | 16.70 ± 15.66 | 48.08 ± 60.76 | 16.11 ± 7.75 | 8.86 ± 4.54 | 7.72 ± 4.35 | 9.58 ± 10.31 |
| 78 | 155.13 ± 78.67 | 35.54 ± 24.12 | 87.56 ± 58.33 | 18.50 ± 6.84 | 9.45 ± 4.01 | 8.39 ± 3.94 | 16.72 ± 9.40 |
| 79 | 45.17 ± 68.27 | 31.18 ± 48.22 | 0.00 ± 0.00 | 1.23 ± 2.85 | 6.25 ± 7.07 | 0.15 ± 0.54 | 5.02 ± 9.81 |
| 79 | 148.18 ± 84.12 | 116.36 ± 53.29 | 0.00 ± 0.00 | 5.66 ± 3.96 | 16.04 ± 7.85 | 0.59 ± 0.92 | 20.35 ± 11.89 |
| 79 | 156.01 ± 76.80 | 118.77 ± 49.75 | 0.00 ± 0.00 | 5.52 ± 3.60 | 17.07 ± 6.80 | 0.40 ± 0.79 | 21.06 ± 13.17 |
| 96 | 123.21 ± 52.18 | 78.34 ± 37.25 | 0.00 ± 0.00 | 12.10 ± 8.01 | 1.61 ± 2.43 | 18.78 ± 11.33 | 11.75 ± 5.95 |
| 96 | 127.54 ± 71.73 | 46.86 ± 43.45 | 0.00 ± 0.00 | 17.32 ± 9.46 | 3.62 ± 3.85 | 30.69 ± 12.06 | 8.14 ± 7.56 |
| 96 | 99.09 ± 67.48 | 34.98 ± 39.39 | 0.00 ± 0.00 | 14.09 ± 8.73 | 2.44 ± 3.45 | 28.09 ± 10.38 | 5.88 ± 6.73 |
| 97 | 29.69 ± 27.71 | 4.67 ± 5.38 | 0.00 ± 0.00 | 11.57 ± 8.96 | 6.41 ± 5.87 | 3.62 ± 6.66 | 2.75 ± 1.87 |
| 97 | 64.82 ± 38.19 | 12.16 ± 6.67 | 0.00 ± 0.00 | 23.11 ± 11.24 | 13.49 ± 6.60 | 16.16 ± 7.41 | 2.61 ± 1.57 |
| 97 | 61.00 ± 30.91 | 10.93 ± 5.72 | 0.00 ± 0.00 | 20.95 ± 9.14 | 11.87 ± 5.45 | 14.63 ± 6.54 | 2.50 ± 1.43 |
| 98 | 24.16 ± 42.32 | 5.94 ± 13.61 | 0.00 ± 0.00 | 5.59 ± 10.96 | 9.41 ± 12.07 | 1.71 ± 4.34 | 9.61 ± 13.85 |
| 98 | 97.42 ± 51.77 | 35.80 ± 16.99 | 0.00 ± 0.00 | 29.42 ± 11.87 | 31.47 ± 12.54 | 11.40 ± 5.31 | 37.83 ± 14.87 |
| 98 | 102.51 ± 46.97 | 36.58 ± 15.44 | 0.00 ± 0.00 | 29.35 ± 10.84 | 30.76 ± 10.91 | 11.26 ± 5.44 | 37.41 ± 13.35 |
| 112 | 27.44 ± 19.21 | 0.00 ± 0.00 | 0.00 ± 0.00 | 5.67 ± 4.20 | 0.35 ± 1.22 | 3.69 ± 2.53 | 19.08 ± 12.50 |
| 112 | 86.84 ± 75.25 | 0.00 ± 0.00 | 0.00 ± 0.00 | 26.13 ± 17.52 | 8.82 ± 7.47 | 14.00 ± 10.42 | 62.75 ± 38.32 |
| 112 | 124.72 ± 73.62 | 0.00 ± 0.00 | 0.00 ± 0.00 | 32.77 ± 16.58 | 12.38 ± 9.49 | 18.96 ± 9.74 | 72.62 ± 32.71 |
| 113 | 238.76 ± 80.38 | 96.99 ± 44.37 | 0.00 ± 0.00 | 10.36 ± 4.40 | 17.08 ± 7.36 | 14.89 ± 4.67 | 101.12 ± 40.82 |
| 113 | 282.91 ± 105.64 | 109.77 ± 43.66 | 0.00 ± 0.00 | 16.26 ± 9.41 | 35.69 ± 18.20 | 20.71 ± 11.01 | 135.14 ± 35.71 |
| 113 | 342.91 ± 68.29 | 125.38 ± 25.54 | 0.00 ± 0.00 | 21.48 ± 7.57 | 43.84 ± 14.35 | 26.24 ± 7.36 | 137.68 ± 28.25 |
| 114 | 20.31 ± 16.63 | 8.31 ± 11.74 | 0.00 ± 0.00 | 6.07 ± 3.07 | 5.07 ± 2.67 | 0.77 ± 1.04 | 1.05 ± 3.43 |
| 114 | 43.49 ± 37.55 | 44.23 ± 29.64 | 0.00 ± 0.00 | 4.54 ± 3.62 | 3.27 ± 2.89 | 0.12 ± 0.40 | 12.74 ± 10.67 |
| 114 | 68.83 ± 35.80 | 52.47 ± 24.92 | 0.00 ± 0.00 | 6.06 ± 3.17 | 4.13 ± 2.67 | 0.16 ± 0.49 | 12.31 ± 7.50 |
| 137 | 0.00 ± 0.00 | 0.00 ± 0.00 | 0.00 ± 0.00 | 0.00 ± 0.00 | 0.00 ± 0.00 | 0.00 ± 0.00 | 0.00 ± 0.00 |
| 137 | 115.79 ± 122.49 | 30.43 ± 25.84 | 0.00 ± 0.00 | 26.08 ± 21.82 | 9.92 ± 9.80 | 13.42 ± 11.80 | 91.49 ± 73.12 |
| 137 | 0.00 ± 0.00 | 0.00 ± 0.00 | 0.00 ± 0.00 | 0.00 ± 0.00 | 0.00 ± 0.00 | 0.00 ± 0.00 | 0.00 ± 0.00 |
| 139 | 0.00 ± 0.00 | 0.01 ± 0.13 | 0.00 ± 0.00 | 0.00 ± 0.00 | 0.00 ± 0.00 | 0.00 ± 0.00 | 0.00 ± 0.00 |
| 139 | 412.85 ± 162.33 | 380.38 ± 118.73 | 0.00 ± 0.00 | 39.95 ± 18.03 | 13.25 ± 8.07 | 16.26 ± 8.51 | 31.94 ± 15.09 |
| 139 | 20.29 ± 37.44 | 21.23 ± 31.96 | 0.00 ± 0.00 | 1.64 ± 3.49 | 0.21 ± 0.96 | 2.60 ± 3.65 | 1.64 ± 3.18 |
| 140 | 0.43 ± 4.18 | 0.10 ± 1.46 | 0.00 ± 0.00 | 0.02 ± 0.45 | 0.02 ± 0.36 | 0.08 ± 0.54 | 0.24 ± 1.89 |
| 140 | 153.46 ± 64.58 | 42.70 ± 18.65 | 0.00 ± 0.00 | 18.48 ± 9.58 | 40.40 ± 13.81 | 22.02 ± 10.70 | 34.27 ± 22.46 |
| 140 | 103.24 ± 109.75 | 42.08 ± 38.14 | 0.00 ± 0.00 | 15.78 ± 13.95 | 11.34 ± 10.96 | 32.60 ± 27.20 | 28.26 ± 27.28 |
| 141 | 5.58 ± 7.92 | 0.00 ± 0.00 | 0.00 ± 0.00 | 0.85 ± 1.50 | 0.07 ± 0.47 | 1.24 ± 2.47 | 3.29 ± 4.11 |
| 141 | 43.38 ± 53.49 | 0.00 ± 0.00 | 0.00 ± 0.00 | 5.38 ± 7.36 | 12.85 ± 13.33 | 3.96 ± 11.05 | 14.07 ± 15.14 |
| 141 | 85.68 ± 85.32 | 0.00 ± 0.00 | 0.00 ± 0.00 | 17.72 ± 14.26 | 26.76 ± 22.74 | 23.34 ± 19.59 | 38.74 ± 30.44 |
| 142 | 180.19 ± 125.83 | 118.08 ± 78.78 | 0.00 ± 0.00 | 11.38 ± 11.86 | 5.60 ± 4.64 | 11.80 ± 13.23 | 33.81 ± 24.23 |
| 142 | 43.00 ± 95.50 | 14.32 ± 39.23 | 0.00 ± 0.00 | 2.20 ± 8.90 | 1.80 ± 6.11 | 0.54 ± 2.28 | 12.46 ± 24.20 |
| 142 | 204.93 ± 124.68 | 102.65 ± 63.55 | 0.00 ± 0.00 | 22.01 ± 16.30 | 14.63 ± 10.03 | 8.62 ± 10.83 | 62.42 ± 38.11 |
| 143 | 190.41 ± 119.48 | 153.64 ± 89.92 | 0.00 ± 0.00 | 9.24 ± 10.91 | 9.89 ± 9.04 | 0.70 ± 3.17 | 11.95 ± 13.14 |
| 143 | 33.48 ± 37.31 | 26.53 ± 28.17 | 0.00 ± 0.00 | 0.42 ± 1.86 | 0.48 ± 2.06 | 0.06 ± 0.60 | 0.91 ± 2.30 |
| 143 | 66.26 ± 87.12 | 42.70 ± 66.42 | 0.00 ± 0.00 | 2.22 ± 5.42 | 3.94 ± 4.01 | 0.14 ± 0.85 | 0.98 ± 2.50 |
| 158 | 11.46 ± 36.95 | 2.23 ± 10.72 | 6.00 ± 19.23 | 0.02 ± 0.16 | 0.00 ± 0.00 | 0.00 ± 0.00 | 2.73 ± 9.68 |
| 158 | 12.05 ± 42.67 | 2.76 ± 13.47 | 3.00 ± 16.35 | 0.23 ± 1.50 | 0.16 ± 1.23 | 0.32 ± 2.04 | 1.51 ± 7.64 |
| 158 | 70.80 ± 90.88 | 19.15 ± 33.12 | 38.31 ± 45.35 | 0.15 ± 0.94 | 0.14 ± 1.16 | 0.21 ± 1.17 | 19.33 ± 24.28 |
| 160 | 36.44 ± 22.66 | 15.20 ± 10.15 | 0.00 ± 0.00 | 2.65 ± 2.24 | 0.37 ± 0.59 | 0.22 ± 0.42 | 19.13 ± 9.32 |
| 160 | 99.60 ± 32.93 | 70.35 ± 22.11 | 0.00 ± 0.00 | 0.88 ± 1.31 | 0.55 ± 1.04 | 1.17 ± 1.34 | 32.20 ± 8.93 |
| 160 | 61.13 ± 55.12 | 46.33 ± 39.51 | 0.00 ± 0.00 | 2.41 ± 3.02 | 1.20 ± 1.97 | 0.25 ± 0.75 | 21.45 ± 13.84 |
| 162 | 170.78 ± 52.03 | 8.79 ± 14.86 | 153.20 ± 38.22 | 0.35 ± 1.38 | 1.10 ± 1.63 | 0.40 ± 1.61 | 13.08 ± 12.76 |
| 162 | 353.15 ± 101.62 | 54.42 ± 18.93 | 230.36 ± 54.37 | 11.35 ± 4.77 | 9.51 ± 4.63 | 30.26 ± 10.47 | 35.19 ± 10.87 |
| 162 | 147.70 ± 66.14 | 16.56 ± 15.95 | 101.23 ± 56.07 | 3.02 ± 3.61 | 3.53 ± 3.98 | 6.58 ± 7.23 | 11.62 ± 8.46 |
| 163 | 0.03 ± 0.27 | 0.00 ± 0.00 | 0.00 ± 0.00 | 0.00 ± 0.00 | 0.04 ± 0.31 | 0.01 ± 0.08 | 0.00 ± 0.00 |
| 163 | 108.07 ± 50.46 | 19.71 ± 11.14 | 0.00 ± 0.00 | 32.83 ± 11.56 | 15.65 ± 6.41 | 31.58 ± 10.18 | 19.04 ± 9.32 |
| 163 | 4.39 ± 9.08 | 0.02 ± 0.25 | 0.00 ± 0.00 | 1.71 ± 3.38 | 1.83 ± 2.89 | 2.02 ± 3.66 | 0.08 ± 0.46 |
| 164 | 2.15 ± 6.25 | 0.39 ± 1.34 | 0.00 ± 0.00 | 0.09 ± 0.44 | 0.22 ± 1.04 | 2.02 ± 4.04 | 1.12 ± 4.83 |
| 164 | 101.67 ± 40.82 | 13.14 ± 6.09 | 0.00 ± 0.00 | 18.72 ± 6.80 | 23.64 ± 9.10 | 45.08 ± 12.63 | 6.12 ± 4.05 |
| 164 | 14.04 ± 18.61 | 1.05 ± 1.93 | 0.00 ± 0.00 | 1.83 ± 2.89 | 3.41 ± 4.55 | 11.11 ± 11.13 | 0.25 ± 0.66 |

| | | | | | | | |
|---|---|---|---|---|---|---|---|
| 165 | 10.88 ± 23.94 | 2.73 ± 8.16 | 0.00 ± 0.00 | 3.30 ± 6.32 | 0.58 ± 1.50 | 5.38 ± 9.34 | 0.11 ± 0.58 |
| 165 | 223.32 ± 79.03 | 76.63 ± 35.00 | 0.00 ± 0.00 | 59.82 ± 14.81 | 20.01 ± 6.42 | 53.01 ± 13.37 | 21.91 ± 10.20 |
| 165 | 84.87 ± 85.21 | 33.75 ± 37.22 | 0.00 ± 0.00 | 25.93 ± 22.61 | 4.53 ± 4.80 | 35.27 ± 25.02 | 2.78 ± 4.29 |
| 166 | 15.29 ± 38.74 | 4.08 ± 12.00 | 0.00 ± 0.00 | 5.70 ± 10.95 | 3.39 ± 7.47 | 5.89 ± 11.70 | 2.07 ± 7.40 |
| 166 | 103.24 ± 41.47 | 18.23 ± 9.42 | 0.00 ± 0.00 | 33.43 ± 11.34 | 30.18 ± 9.61 | 22.46 ± 7.34 | 3.57 ± 8.25 |
| 166 | 89.17 ± 89.27 | 32.09 ± 37.87 | 0.00 ± 0.00 | 27.22 ± 19.74 | 13.51 ± 11.83 | 14.95 ± 12.20 | 14.41 ± 21.13 |
| 167 | 47.26 ± 90.26 | 23.35 ± 36.64 | 0.00 ± 0.00 | 11.55 ± 19.83 | 8.21 ± 15.24 | 3.65 ± 7.34 | 25.39 ± 35.82 |
| 167 | 268.97 ± 86.83 | 95.26 ± 24.95 | 0.00 ± 0.00 | 34.70 ± 10.14 | 38.62 ± 12.43 | 14.39 ± 6.25 | 98.43 ± 23.78 |
| 167 | 217.95 ± 133.60 | 84.48 ± 41.25 | 0.00 ± 0.00 | 28.54 ± 16.22 | 35.78 ± 20.83 | 16.95 ± 11.54 | 79.36 ± 35.35 |
| 169 | 51.01 ± 95.51 | 66.36 ± 116.37 | 0.00 ± 0.00 | 7.99 ± 13.86 | 6.80 ± 11.80 | 6.99 ± 9.87 | 19.90 ± 24.67 |
| 169 | 82.92 ± 28.95 | 49.11 ± 15.76 | 0.00 ± 0.00 | 2.46 ± 3.52 | 6.46 ± 3.54 | 5.94 ± 3.87 | 25.99 ± 9.81 |
| 169 | 59.16 ± 36.97 | 18.33 ± 16.41 | 0.00 ± 0.00 | 5.42 ± 5.16 | 8.19 ± 5.71 | 8.51 ± 4.85 | 25.86 ± 11.34 |

# References


(S1) Teriete, P.; Banerji, S.; Noble, M.; Blundell, C. D.; Wright, A. J.; Pickford, A. R.; Lowe, E.; Mahoney, D. J.; Tammi, M. I.; Kahmann, J. D. Structure of the regulatory hyaluronan binding domain in the inflammatory leukocyte homing receptor CD44. *Molecular Cell* **2004**, *13*, 483–496.

(S2) Banerji, S.; Wright, A. J.; Noble, M.; Mahoney, D. J.; Campbell, I. D.; Day, A. J.; Jackson, D. G. Structures of the Cd44–hyaluronan complex provide insight into a fundamental carbohydrate-protein interaction. *Nature Structural & Molecular Biology* **2007**, *14*, 234–239.

(S3) Skelton, T. P.; Zeng, C.; Nocks, A.; Stamenkovic, I. Glycosylation provides both stimulatory and inhibitory effects on cell surface and soluble CD44 binding to hyaluronan. *The Journal of Cell Biology* **1998**, *140*, 431–446.

(S4) Reitsma, S.; Slaaf, D. W.; Vink, H.; van Zandvoort, M. A.; oude Egbrink, M. G. The endothelial glycocalyx: composition, functions, and visualization. *Pflügers Archiv-European Journal of Physiology* **2007**, *454*, 345–359.

(S5) Yang, C.; Cao, M.; Liu, H.; He, Y.; Xu, J.; Du, Y.; Liu, Y.; Wang, W.; Cui, L.; Hu, J. The high and low molecular weight forms of hyaluronan have distinct effects on CD44 clustering. *Journal of Biological Chemistry* **2012**, *287*, 43094–43107.

(S6) Xu, G.-K.; Qian, J.; Hu, J. The glycocalyx promotes cooperative binding and clustering of adhesion receptors. *Soft matter* **2016**, *12*, 4572–4583.

(S7) Lindorff-Larsen, K.; Piana, S.; Palmo, K.; Maragakis, P.; Klepeis, J. L.; Dror, R. O.; Shaw, D. E. Improved side-chain torsion potentials for the Amber ff99SB protein force field. *Proteins: Structure, Function, and Bioinformatics* **2010**, *78*, 1950–1958.

(S8) Kirschner, K. N.; Yongye, A. B.; Tschampel, S. M.; González-Outeiriño, J.; Daniels, C. R.; Foley, B. L.; Woods, R. J. GLYCAM06: a generalizable biomolecular force field. Carbohydrates. *Journal of Computational Chemistry* **2008**, *29*, 622–655.

(S9) Jorgensen, W. L.; Chandrasekhar, J.; Madura, J. D.; Impey, R. W.; Klein, M. L. Comparison of simple potential functions for simulating liquid water. *The Journal of chemical physics* **1983**, *79*, 926–935.

(S10) Dang, L. X.; Rice, J. E.; Caldwell, J.; Kollman, P. A. Ion solvation in polarizable water: molecular dynamics simulations. *Journal of the American Chemical Society* **1991**, *113*, 2481–2486.

(S11) Moore, D. S. *The basic practice of statistics*; WH Freeman New York: New York, USA, 2007; Vol. 2.

(S12) Jamison II, F. W.; Foster, T. J.; Barker, J. A.; Hills Jr, R. D.; Guvench, O. Mechanism of binding site conformational switching in the CD44–hyaluronan protein–carbohydrate binding interaction. *Journal of Molecular Biology* **2011**, *406*, 631–647.
